# Late date of human arrival to North America: Continental scale differences in stratigraphic integrity of pre-13,000 BP archaeological sites

**DOI:** 10.1371/journal.pone.0264092

**Published:** 2022-04-20

**Authors:** Todd A. Surovell, Sarah A. Allaun, Barbara A. Crass, Joseph A. M. Gingerich, Kelly E. Graf, Charles E. Holmes, Robert L. Kelly, Marcel Kornfeld, Kathryn E. Krasinski, Mary Lou Larson, Spencer R. Pelton, Brian T. Wygal

**Affiliations:** 1 Department of Anthropology, University of Wyoming, Laramie, Wyoming, United States of America; 2 Museum of the North, University of Alaska, Fairbanks, Alaska, United States of America; 3 Department of Anthropology, Ohio University, Athens, Ohio, United States of America; 4 Department of Anthropology, National Museum of Natural History, Smithsonian Institution, Washington, D.C., United States of America; 5 Center for the Study of the First Americans, Department of Anthropology, Texas A&M University, College Station, Texas, United States of America; 6 Department of Anthropology, University of Alaska Fairbanks, Fairbanks, Alaska, United States of America; 7 Department of Anthropology, Adelphi University, Garden City, New York, United States of America; 8 Office of the Wyoming State Archaeologist, University of Wyoming, Laramie, Wyoming, United States of America; University of Michigan, UNITED STATES

## Abstract

By 13,000 BP human populations were present across North America, but the exact date of arrival to the continent, especially areas south of the continental ice sheets, remains unclear. Here we examine patterns in the stratigraphic integrity of early North American sites to gain insight into the timing of first colonization. We begin by modeling stratigraphic mixing of multicomponent archaeological sites to identify signatures of stratigraphic integrity in vertical artifact distributions. From those simulations, we develop a statistic we call the Apparent Stratigraphic Integrity Index (ASI), which we apply to pre- and post-13,000 BP archaeological sites north and south of the continental ice sheets. We find that multiple early Beringian sites dating between 13,000 and 14,200 BP show excellent stratigraphic integrity. Clear signs of discrete and minimally disturbed archaeological components do not appear south of the ice sheets until the Clovis period. These results provide support for a relatively late date of human arrival to the Americas.

## Introduction

No consensus has been reached among archaeologists about the date of initial human arrival to the Americas, but all agree that human populations were distributed across the North American continent by 13,000 BP, as evidenced by fluted Clovis projectile points and associated artifacts from surface and buried contexts [1–5]. Before 13,000 BP, the clearest evidence for human presence comes from eastern Beringia, the unglaciated portions of Alaska and the Yukon Territory [e.g, 6–13]. Evidence for humans south of the Laurentide and Cordilleran ice sheets prior to 13,000 BP remains sparse and controversial despite more than a century of fieldwork in the region [e.g., 14–26]. Archaeologists widely accept the critical appraisal of artifacts from firmly dated pre-13,000 BP contexts as a valid means of evaluating claims for early human presence in the Americas [27–32]. When artifacts are found in buried contexts pre-dating 13,000 BP, there are at least two possible explanations for their occurrence—humans were present before 13,000 BP, or humans were not present but younger artifacts have intruded into older sedimentary contexts. Many debates regarding the peopling of the Americas can be reduced to distinguishing between these two possibilities.

Unambiguous association between artifacts and the strata from which they are derived has been a hallmark of establishing the antiquity of humans in the Americas since the earliest days of American archaeology [27,31], yet archaeological tools for evaluating these associations remain crude. Here, we develop a simple means of evaluating the stratigraphic integrity of association between buried archaeological occupations and dated strata and then evaluate the early North American archaeological record using existing provenience data to distinguish between an early or late arrival. The classic Clovis-first model posits that humans arrived in eastern Beringia sometime prior to the Clovis period and breached the continental ice sheets around or just before 13,000 BP [33,34]. If so, the oldest evidence for intact occupations should first occur in eastern Beringia and then appear south of the ice sheets in Clovis times. If there was a widespread pre-13,000 BP population south of the ice sheets, there should be clear evidence for it in the form of unambiguous occupations from archaeological contexts reliably pre-dating 13,000 BP.

## Modeling stratigraphic mixing

To develop expected differences between vertically mixed and unmixed multicomponent sites, we created a simulation for R v. 4.1.1 [35] that combines a depositional history, an occupational history, and a disturbance model (S1 File). For our simulations, we hold depositional and occupational history constant, but our model can be customized to accommodate any such history. In our simulation, sediments accumulate over 18,000 years at a constant rate of 0.1 mm per year. Seven occupations take place, each spaced 2,000 years apart with the first occurring at 12,500 BP and the last at 500 BP. During each occupation, 500 artifacts are deposited on the ground surface at the time, and each artifact is assigned a random arbitrary horizontal provenience. Each year, all artifacts are moved randomly up or down a fixed distance determined by a maximum dispersal rate and depth. If any artifacts breach the ground surface, they are placed on the surface that exists at the time. It is assumed that artifacts closer to the current ground surface are more susceptible to post-depositional disturbance and displacement and therefore should move more than those at greater depth. Following from that assumption, we modeled actual artifact dispersal rates (*r*) as a logistic function of depth (*d*) and maximum dispersal rate (*r*_*max*_):

$$r=\frac{r_{max}}{0.777+e^{-3(0.5-d)}}$$ (1)

This function is shown graphically in S1 Fig. If the maximum dispersal rate is set to 1 mm per year, for example, an artifact 5 cm beneath the ground surface will move 0.97 mm each year up or down. An artifact 50 cm beneath the surface will move 0.56 mm per year. An artifact buried 1 m in depth will move 0.19 mm, and one at 2 m of depth will move around 0.01 mm per year. We do not mean to imply that this function can be used to describe all archaeological cases, but it is intended to be a reasonable approximation of the relative degree to which artifacts at different depths are subject to post-depositional disturbance processes originating from the ground surface such as bio- and cryoturbation.

**Fig 1:**
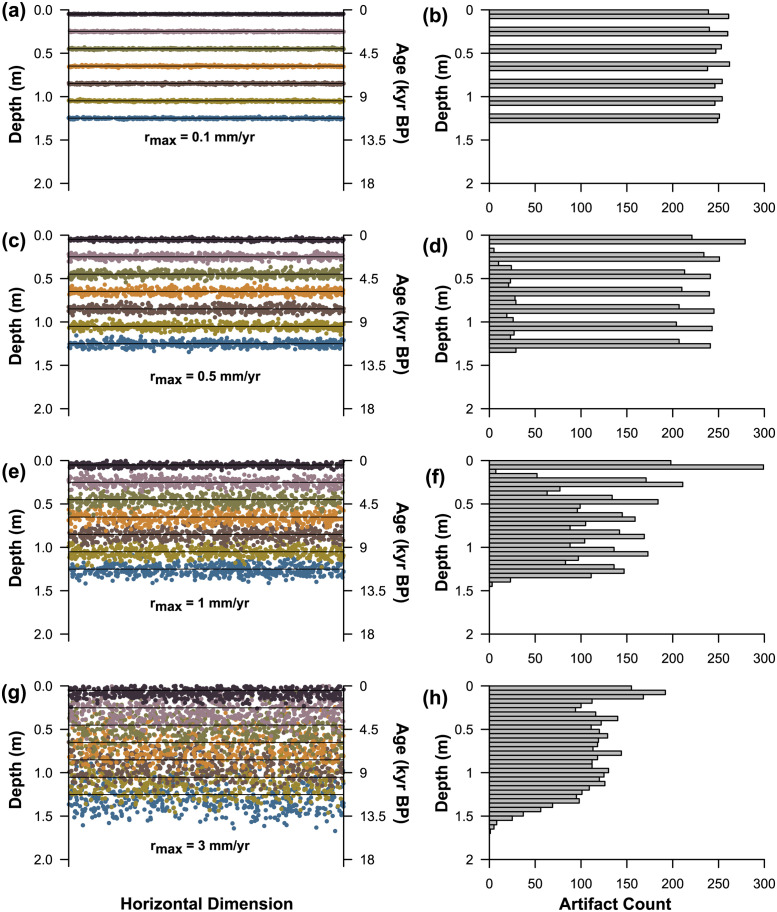
Simulations of stratigraphic mixing of a multicomponent archaeological site shown as backplots (left) and histograms of artifact elevation (right) while varying the maximum rate of artifact dispersal (r_max_). Horizontal black lines on backplots show the original stratigraphic position of occupations before post-depositional disturbance. (a&b) r_max_ = 0.1 mm/yr. (c&d) r_max_ = 0.5 mm/yr. (e&f) r_max_ = 1.0 mm/yr. (g&h) r_max_ = 3.0 mm/yr.

In Fig 1, we illustrate four outputs of the simulation while varying maximum dispersal rate (*r*_*max*_). Specifically, we show resulting backplots and vertical density histograms. With *r*_*max*_ set to a low rate of 0.1 mm per year, each occupation is vertically discrete with culturally sterile zones separating occupations. Artifacts have not moved far beyond the surfaces on which they were originally deposited. When viewed as a vertical density histogram, the minimally disturbed case exhibits a very peaked, multimodal distribution with clear stratigraphic integrity (Fig 1a and 1b).

When *r*_*max*_ is set to a much higher rate (e.g., 3 mm per year), individual occupations are not stratigraphically discrete and become visibly mixed (Fig 1g and 1h). As others have found, with significant mixing archaeological distributions become vertically homogenized [36,37], although artifacts from different occupations generally maintain their stratigraphic order. In this situation, it would be challenging to assign individual artifacts to specific occupations. It would also be difficult using vertical distributions alone to determine how many occupations occurred. In the highly mixed simulation, artifacts can be found well above and below the surfaces on which they were deposited. At depth, artifact densities slowly decline to zero. Near the surface, a sharp mode in artifact count is evident because the most recent occupation has had the least time to be dispersed leaving somewhat high densities at the uppermost levels. When viewed as a vertical density histogram, the overall distribution shows no gaps, and little multimodality. Overall, the vertical density distribution has a down-skewed appearance. The two intermediate cases show intermediate attributes of the minimally and maximally mixed cases, with varying degrees of multimodality (Fig 1c–1f).

**Fig 2:**
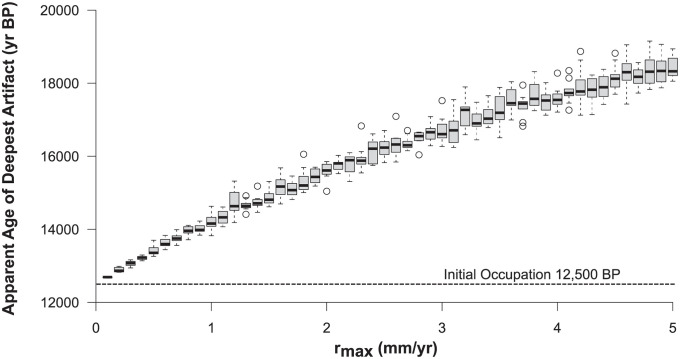
The relationship between simulated rates of artifact dispersal (r_max_) and the apparent age of the deepest artifact. Dashed line shows the age of the initial occupation at 12,500 BP.

To illustrate the effect of increased mixing on the apparent age of the deepest artifacts in this system, we varied *r*_*max*_ from 0.1 to 5 mm per year and ran ten iterations of each model to determine how the apparent age of the deepest artifact increases with greater disturbance (Fig 2). Apparent age in this context refers to the age of the sediments from which the deepest artifact was recovered. With increased mixing, artifacts move progressively downward into older sediments producing the false impression of the presence of humans on a site hundreds to thousands of years prior to the initial occupation.

### ASI: Apparent Stratigraphic Integrity Index

In our simulated multicomponent archaeological cases, the degree of vertical mixing is clearly reflected by changes in vertical artifact distributions. We developed a statistic that we call the Apparent Stratigraphic Integrity Index (ASI) that captures this variability. The use of the term “apparent” is because frequently reoccupied but minimally disturbed sites could theoretically exhibit similar stratigraphic artifact distributions to highly disturbed sites (S2 File; S2 Fig). Likewise, the vertical artifact distribution from a highly disturbed site could exhibit properties typical of a minimally disturbed locality. To determine with greater certainty the extent to which artifacts have moved vertically in an actual archaeological site would require additional kinds of data (e.g., systematic refitting). All things being equal, however, we expect minimally disturbed multicomponent sites to show dramatic changes in artifact density from stratum to stratum (Fig 1a and 1b) and heavily disturbed sites to show gradual changes in density from level to level (Fig 1g and 1h).

The ASI is based on changes in the artifact frequency between adjacent excavation levels. In general statistical nomenclature, this statistic could be called the relative mean absolute successive difference and is similar to the unstandardized mean absolute successive difference (MASD) [38]. Before calculating the ASI, consecutive levels with zero artifacts are collapsed to single zero values. After that transformation, the calculation of ASI uses the mean absolute change in artifact frequency (*f*) between all adjacent levels divided by two times the mean artifact frequency for all levels:

$$ASI=\frac{\sum_{i=1}^{n-1}\left|f_{i}-f_{i+1}\right|}{2\frac{\sum_{i=1}^{n}f_{i}}{n}}$$ (2)

For most cases, the ASI varies between zero and one with higher values implying greater stratigraphic integrity. Consecutive sterile levels are reduced to a single zero value because if left in, they would result in a lower ASI value when they should theoretically have little bearing on the question of stratigraphic integrity. To calculate an ASI for a site, an excavation unit, or any systematically excavated area, the function requires an array of artifact counts sorted by excavated level (S1 File). One disadvantage of the ASI as we have constructed it is that its value can vary depending upon an arbitrary choice of thickness of excavation levels or elevation bins. For our application, however, due to the nature of the available data, we use artifact counts from standardized 5 cm levels. This choice is also justified because the use of 5 cm levels is fairly standard practice in excavations of hunter-gatherer archaeological sites. It is important to note that comparison of ASI values among sites is best done using standardized level thicknesses, since coarse units of inquiry (i.e., thicker levels) will exhibit lower ASI values than fine ones.

**Fig 3:**
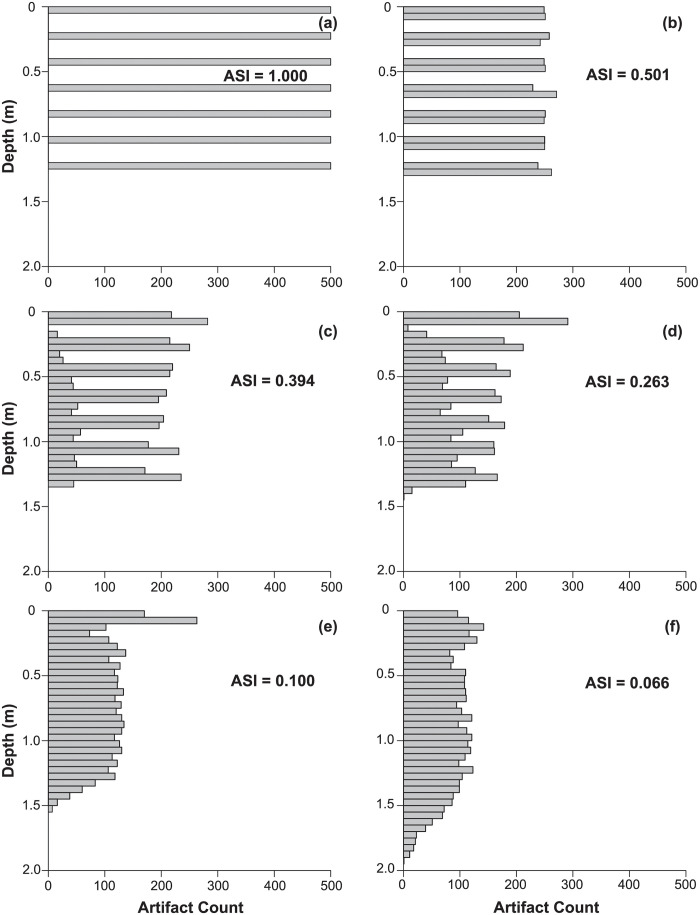
ASI values for six simulated artifact distributions from no mixing (a) to severe mixing (f).

In Fig 3, we show ASIs for six simulated vertical artifact distributions. Generally speaking, highly homogenized vertical distributions have low ASI values, but those with gaps representing sterile or low density strata separating occupations exhibit high ASIs. In the examples shown, relatively intact distributions with little mixing have ASIs from 0.4 to 1.0. Highly mixed sites tend to show values less than 0.3.

In addition to sensitivity to bin width (or the width of excavated levels), the ASI is also affected by small sample size (S3 Fig). For several levels of disturbance, we randomly sampled between ten and 3,500 artifacts from simulated sites and calculated the resulting ASIs (S2 File). This exercise showed that low density sites with very few artifacts will exhibit inflated ASI values. Sites that show less than a mean of approximately 30 artifacts per level will appear more intact than they actually are when measured by ASI. This problem reinforces the idea that it is difficult to determine the stratigraphic integrity of any archaeological site with a small assemblage. On the opposite end of the spectrum, ASI values should be most reliable for sites with high artifact densities.

## The apparent stratigraphic integrity of paleoindian sites

**Fig 4:**
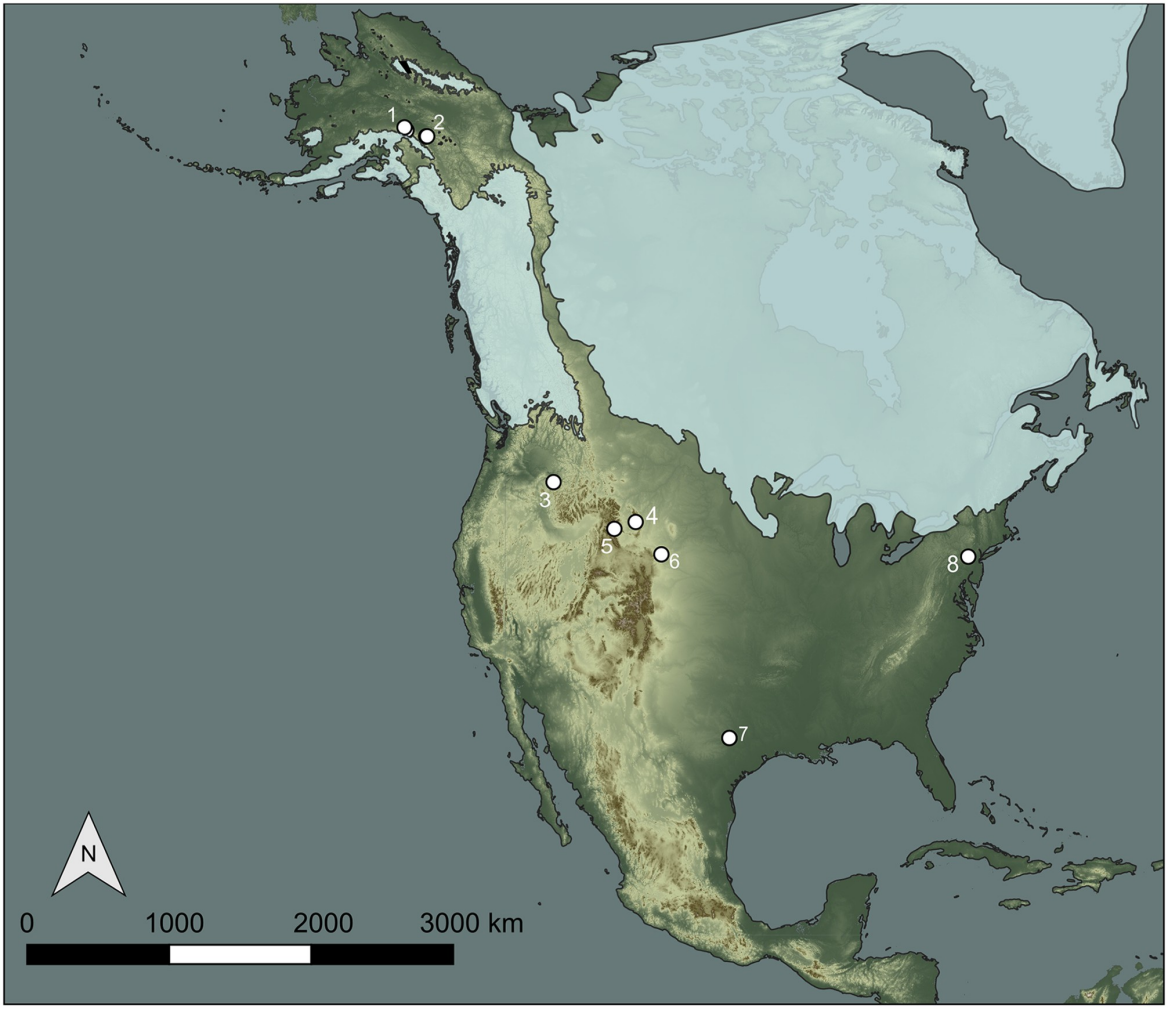
Map of sites used in this study. (1) Dry Creek and Owl Ridge, (2) Holzman South, Swan Point, and Broken Mammoth, (3) Cooper’s Ferry, (4) Alm Shelter; (5) Helen Lookingbill; (6) Hell Gap; (7) Gault and Debra L. Friedkin, (8) Shawnee-Minisink. Light blue polygons show the estimated extent of the continental ice sheets at 14,900 BP from Dalton et al. [57]. Digital elevation model of North America is from the USGS Global Multi-resolution Terrain Elevation Dataset [58].

We compiled vertical density data from a series of North American multicomponent sites that contain Paleoindian occupations (Fig 4, S2 File). No permits were required for the described study, which complied with all relevant regulations. Artifact density data are either first reported here or were taken from published literature. The sample includes eight sites with components argued to pre-date 13,000 BP, five from eastern Beringia and three from south of the ice sheets. Beringian pre-13,000 BP sites include Broken Mammoth [7,39,40], Dry Creek [10,12], Holzman South [9,13], Swan Point [6,8], and Owl Ridge [41,42]. All Beringian sites occur within a 200 km reach of the Tanana River valley and its tributaries in eastern Alaska and are buried in Pleistocene loess. The oldest components in all of these sites are argued to date between ca. 13,000 and 14,200 BP. Pre-13,000 BP sites south of the ice sheets include Cooper’s Ferry [19,43,44], Debra L. Friedkin [18,45], and Gault [46,47]. The oldest occupations at these sites are hypothesized to date to at least 15,500 to 18,500 BP [18,19,45,47]. We considered the inclusion of other potential pre-Clovis sites [e.g., 16,20,21,48], but appropriate data were not available, the site was insufficiently buried, or artifact counts were too low. Four multicomponent sites with Paleoindian occupations post-dating 13,000 BP are also part of the sample. They include Alm Shelter [49], Helen Lookingbill [50], Hell Gap, Locality I [51–53], and Shawnee-Minisink [54–56]. Importantly, Shawnee-Minisink has a Clovis component, and due to the nature of the excavations at Shawnee (S2 File), we only have vertical artifact distribution data for the strata surrounding and including the Clovis component.

To generate vertical density profiles, for all but two sites (Gault and Friedkin), we binned the elevations of piece-plotted artifacts into 5 cm levels after adjusting for stratigraphic tilt. When using this method, we omitted artifacts recovered from screens due to lower precision provenience. To account for sloping stratigraphy when present, we isolated portions of sites where at least one planar artifact concentration could be discerned within a three-dimensional backplot and fit a plane through it using multiple linear regression (S4 Fig). We then transformed absolute artifact elevations to relative elevations above or below that plane. If no stratigraphic slope could be discerned, we binned artifacts into 5 cm levels using elevations. We do not have piece-plot data for Gault and Debra L. Friedkin, so we compiled vertical density profiles from previously published 5 cm level counts [18,47] and could not account for stratigraphic tilt.

**Fig 5:**
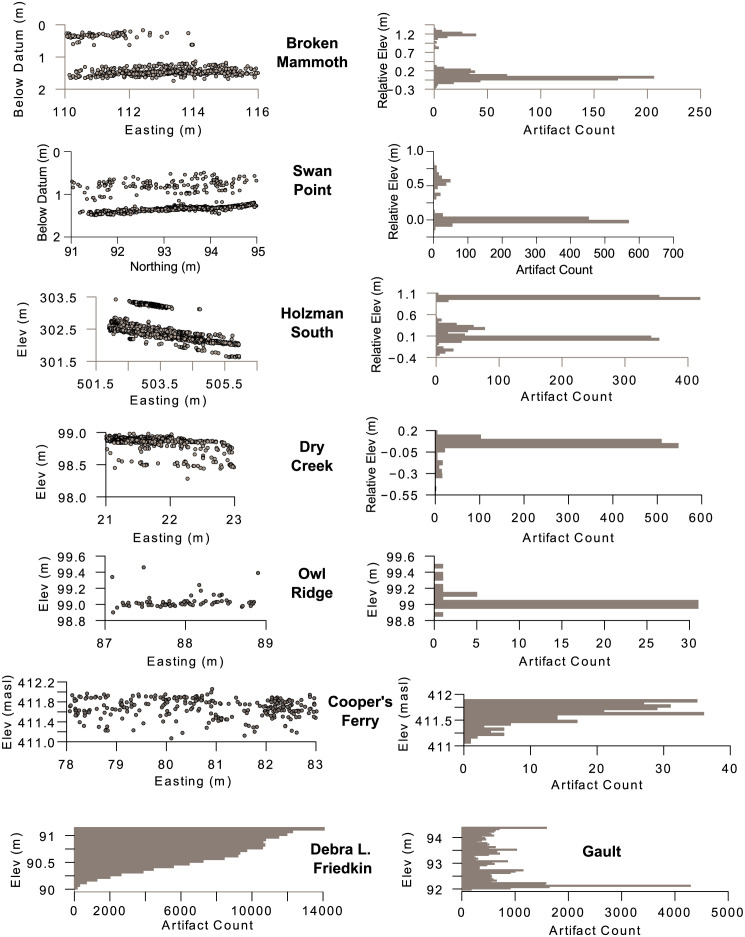
Backplots and/or vertical density histograms for all pre-13,000 BP archaeological sites in the study.

All five eastern Beringian sites show multiple stratigraphically discrete archaeological components (Fig 5). Backplots of these Alaskan sites show easily defined planar archaeological components separated by low density or sterile stratigraphic units. Vertical density histograms are unambiguously multimodal. The pre-13,000 BP sites south of the continental ice sheets display very different patterns. Cooper’s Ferry and Friedkin exhibit similar density profiles to each other, even though artifact densities between the two sites differ by three orders of magnitude. Both sites exhibit traits expected of vertically mixed deposits with smoothed and homogenized distributions slowly tailing off with depth. The profile from Gault, however, shares traits with both the Beringian and southern pre-Clovis sites. It is clearly multimodal, but it lacks clear gaps or sterile zones between archaeological components. To what extent the Gault profile is affected by sloping stratigraphy, we do not know.

**Fig 6:**
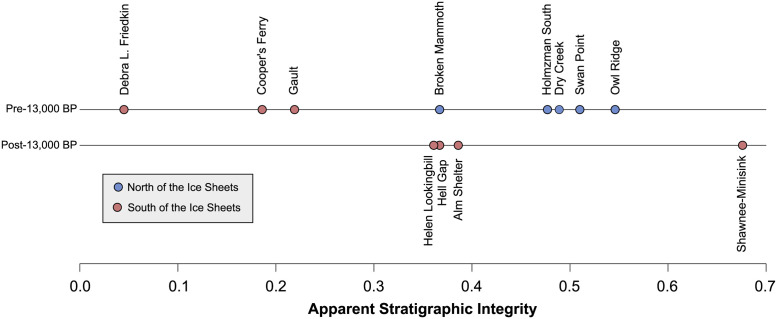
ASI values for all pre- and post-13,000 BP sites in the study.

We calculated ASI values for all pre- and post-13,000 BP sites (Table 1; Fig 6). There are clear differences between the pre-13,000 BP sites north and south of the continental glaciers. All five Beringian sites show relatively high levels of apparent stratigraphic integrity with ASI values ranging from 0.367 at Broken Mammoth to 0.546 at Owl Ridge. The Owl Ridge ASI is likely inflated due to relatively low artifact densities, but its deepest component is stratigraphically discrete. Still, all early Beringian sites cluster together. Early sites south of the ice sheets also form a cluster, but on the low end of the ASI scale. The Debra L. Friedkin site has the lowest ASI of any observed site at 0.045. The Gault site shows the highest apparent stratigraphic integrity of these three at 0.219. Cooper’s Ferry has an intermediate value, which could be inflated due to low artifact counts. Differences between the mean ASIs of early northern and southern sites are significant (Welch t-test, t = 5.359, df = 3.303, two-tailed p = 0.01). While the post-13,000 BP sites south of the ice sheets show extremely variable ASI from 0.343 (Helen Lookingbill) to 0.676 (Shawnee Minisink) (S5–S8 Figs), they are higher than all supposed pre-Clovis sites south of the ice sheets. They are also not of demonstrably different magnitude than the pre-13,000 BP sites north of the ice sheets (Welch t-test, t = 0.418, df = 3.87, two-tailed p = 0.698), but they are significantly greater than the southern pre-13,000 BP sites (Welch t-test, t = 3.096, df = 4.863, two-tailed p = 0.028).

**Table 1:** ASI values and mean items per 5 cm level for all sites in the study.

Site	Mean Items Per 5 cm Level^a^	ASI
Alm Shelter	14.6	0.386
Broken Mammoth	27.8	0.367
Cooper’s Ferry	14.1	0.186
Debra L. Friedkin	7150	0.045
Dry Creek	88.9	0.489
Gault	752	0.219
Helen Lookingbill	74.9	0.343
Hell Gap	12.4	0.367
Holzman South	78.5	0.477
Owl Ridge	5.3	0.546
Shawnee-Minisink	93.3	0.676
Swan Point	59.3	0.510

^a^Mean counts are calculated after removal of consecutive sterile levels.

## Discussion

Integrity of association between artifacts and the dated stratigraphic contexts from which they derive is a baseline standard for establishing the antiquity of humans in the Americas. Our systematic evaluation of some of the earliest buried archaeological sites in North America supports a relatively late arrival of humans to areas south of the continental ice sheets. In our sample, the oldest sites demonstrating relatively unmixed and discrete occupations prior to 13,000 BP all occur in eastern Beringia, which is noteworthy considering these sites are located in an area commonly affected by cryoturbation [12,42]. The archaeologists working at these Alaskan sites were aware of and can identify the problems that bioturbation, solifluction, and other phenomena can bring to the excavation, data recovery, and its analysis and interpretation; excavators frequently consulted with pedologists and Quaternary geologists throughout. The Alaskan sites analyzed here contained unambiguous minimally disturbed archaeological components. For example, at Broken Mammoth, Swan Point, and several other sites the stratigraphy was straightforward and the radiocarbon dates were in correct order [59]. At Dry Creek [12] and Broken Mammoth [60,61], the oldest cultural component occurred beneath a thin sand layer that physically separated that component from the younger components. At the Holzman site, up to 20 cm of culturally sterile loess deposits separate components 4 from component 5a and sterile bands of sand deposits separate components 5a from 5b [9,13].

All of the pre-13,000 BP sites south of the continental ice sheets display patterns of significant mixing. South of the ice sheets, the first evidence for a discrete occupation is from the Clovis period at the Shawnee-Minisink site, which given its latitude and age, could have also experienced cryoturbation. Stratigraphically discrete occupations regularly occur in the archaeological record from the Clovis period onward. Furthermore, Shawnee-Minisink is one of several Clovis sites to exhibit a stratigraphically discrete cultural occupation [e.g., 62–69]; this is a trait clearly associated with the Clovis complex and not associated with any sites pre-dating Clovis in the New World, except those in Beringia. To some extent, data availability for the oldest purported sites in the Americas undermined this study by limiting sample size of site south of the Canadian ice sheets. Sites claimed to be older than 13,000 BP are few and data supporting their status as sites have been poorly disseminated. Given the status of available data regarding these sites, we must question whether there are any sites in the Americas south of the ice sheets that exhibit an unambiguous and stratigraphically discrete cultural occupation with sufficient numbers of artifacts of clear human manufacture.

One site that might be argued to meet those criteria is Monte Verde, Chile [24,70,71], but many researchers question whether most objects from the MV-II Pleistocene peat bog are truly artifacts, especially the organic items. There are only about six items that satisfy most critics as undoubtedly of human manufacture [27,72]. Other sites, like Page-Ladson and Paisley Caves have very small numbers of artifacts, such that evaluating stratigraphic integrity is challenging [73,74]. Even where large numbers of questionable artifacts are argued to be present as at Chiquihuite Cave or Pedra Furada, they do not occur in discrete identifiable archaeological components [14,75].

Another site that might preserve a stratigraphically discrete occupation below Clovis is Cactus Hill, Virginia, which is argued to have an archaeologically sterile zone separating the Clovis and pre-Clovis levels [20,76,77], but data demonstrating stratigraphic separation have not been published. Furthermore, no archaeologically sterile levels separating the Clovis and pre-Clovis components are evident in the vertical artifact distribution data that have been published [20]. Because excavated levels at Cactus Hill were not dug in uniform thicknesses and no provenience data for piece-plotted artifacts are available, it is difficult to compare ASIs from Cactus Hill to other sites. Nonetheless, using vertical artifact distribution data from Areas A/B and B of the site [20], it is possible to calculate ASIs, and like other pre-Clovis sites south of the ice sheets, Cactus Hill does not preserve discrete archaeological components, and ASIs generally fall in the range of 0.156 to 0.309 (S9 Fig). Furthermore, artifact counts gradually decline with depth (S9 Fig). Both findings suggest that Cactus Hill has likely experienced some mixing, and the excavators have noted that “downdrift from upper levels” has affected lower levels of the site [20].

All three of the pre-Clovis sites outside of Alaska included in this study have been argued to be stratigraphically intact, and questions have been raised about two of them [18,19,45,47,78,79]. Our analysis supports the hypothesis that mixing has affected these sites, and furthermore, we note that it would be a simple matter to establish whether that is in fact the case. The ideal way to do so would be systematic refitting of chipped stone artifacts with the goal of searching for refits between components to determine to what extent items are moving vertically in these sites. There is a long tradition of such studies in archaeology, including Paleoindian archaeology both north and south of the ice sheets [39,42,46,80–85]. If little vertical distance separates refitting and conjoining artifacts, it might be shown that artifacts have not moved significantly. Notably, a stratigraphically restricted study of Clovis technology from a different excavation area at the Gault site than the one analyzed herein found that artifacts were moving vertical distances of at least 19 cm from a sample of only 27 refits [46]. Examination of stratigraphic differences in artifact size, long axis dip/inclination, lithic raw materials, technology, or burning of artifacts could provide independent evidence for stratigraphic integrity. Of great importance is the presence of cultural features in sealed stratigraphic units associated with unambiguous occupation surfaces marked by sufficient artifact counts to allow their identification. In short, there is no evidence for a stratigraphically discrete archaeological occupation with large numbers of artifacts before 13,000 BP in the New World except those in Beringia.

These observations provide support for the hypothesis that the first arrival of humans to areas south of the Laurentide and Cordilleran glaciers occurred near in time to 13,000 BP. It is possible humans colonized the New World thousands of years before 13,000 BP, but if they did, they should have produced stratigraphically discrete occupation surfaces, some of which would be expected to have large numbers of artifacts. That they did so in Beringia but failed to do so south of the continental glaciers suggests that either there was something fundamentally different about pre-Clovis human behavior and/or geomorphology south of the ice sheets or that the evidence indicating the presence of humans south of the ice sheets has been misinterpreted. At a minimum, it shows that when stratigraphically discrete occupations are not present, additional studies must be performed to demonstrate that stratigraphic integrity of association between artifacts and dated strata exists.

## Conclusion

The oldest evidence for archaeological sites in the New World with large numbers of artifacts occurring in discrete and minimally disturbed stratigraphic contexts occur in eastern Beringia between 13,000 and 14,200 BP. South of the ice sheets, the oldest such sites occur in association with the Clovis complex. If humans managed to breach the continental ice sheets significantly before 13,000 BP, there should be clear evidence for it in the form of at least some stratigraphically discrete archaeological components with a relatively high artifact count. So far, no such evidence exists. These findings support the hypothesis that the first human arrival to the New World occurred by at least 14,200 BP in Beringia and by approximately 13,000 BP in the temperate latitudes of North America. Strong evidence for human presence before those dates has yet to be identified in the archaeological record.

## Supporting information

S1 Fig Dispersal rate function used in mixing simulation. Shown for *r*_*max*_ = 1 mm/yr.
(PDF)

S2 Fig Results of a simulation illustrating a case of low ASI and high stratigraphic integrity illustrated as a backplot (left) and vertical density histogram (right). In this model, occupation intensity gradually increases over time and no vertical mixing occurs. In the backplot, artifacts are colored by occupation.
(PDF)

S3 Fig Average number of artifacts per level vs. ASI for simulated multicomponent site while varying the maximum rate of artifact dispersal (r_max_). (PDF)

S4 Fig Example of method used to correct artifact elevations for stratigraphic tilt. Three-dimensional scatterplot of artifacts from Holzman South with a plane fit by multiple linear regression to component 5a.
(PDF)

S5 Fig Backplot and vertical density histogram for Locality I of the Hell Gap site, Wyoming for N 1481 to 1482 m and E 1294.5 to 1296.1 m. (PDF)

S6 Fig Backplot and vertical density histogram Alm Shelter, Wyoming for N98 to 99 and E 99 to 100. (PDF)

S7 Fig Backplot and vertical density histogram for the Helen Lookingbill site, Wyoming for N 1014 to 1015 m and E 974 to 976 m. (PDF)

S8 Fig Backplot and vertical density histogram of the Clovis component from the Shawnee-Minisink site, Pennsylvania for N 154.89 4 to 158.374 m and E 152.37 to 155.39 m. (PDF)

S9 Fig Artifact counts by level for four areas of the Cactus Hill site. a. Block B, Unit 16; b. Block B, Unit 17; c. W165 N100; d. W115 N70. Excavation levels at Cactus Hill were not dug in uniform thicknesses.
(PDF)

S1 Table Chipped stone artifact counts by 5 cm level for unit N98 E99 of Alm Shelter. (PDF)

S2 Table Artifact and bone counts by 5 cm level for N 96 to 100 m and E 110 to 199 m of the Broken Mammoth site. Relative elevation in the distance above or below a plane fit through the LP component.
(PDF)

S3 Table Artifact and bone counts by 5 cm level for stratum LU3 of Area A of the Cooper’s Ferry site. (PDF)

S4 Table Debitage and tool count by 5 cm level for Block A of the Debra Friedkin site. (PDF)

S5 Table Chipped stone artifact counts by 5 cm level for N 14 to 16 m and E 21 to 23 m of the Dry Creek site. Relative elevation in the distance above or below a plane fit through Component 2.
(PDF)

S6 Table Counts of flakes by 5 cm level from Area 15 of the Gault site. (PDF)

S7 Table Chipped stone artifact and bone counts by 5 cm level for N 1014 to 1015 m and E 974 to 976 m from the Helen Lookingbill site. Relative elevations are the distance above or below a plane fit through all artifacts between elevations 98.7 and 99.1 m.
(PDF)

S8 Table Counts of chipped stone artifacts, ocher, and bone by 5 cm level from N 1481 to 1482 m and E 1294.5 and 1296.1 m of Locality I of the Hell Gap site. (PDF)

S9 Table Artifact and bone counts by 5 cm level for N 185 to 192 m and E 502 to 506 m from the Holzman South site. Relative elevations are the distance above or below a plane fit through artifacts from Component 5a.
(PDF)

S10 Table Artifact counts by 5 cm level for N 87 to 89 m and E 110 to 111 m from the Owl Ridge site. (PDF)

S11 Table Artifact counts by 5 cm level for N 158.9 to 158.4 m and E 152.4 to 155.4 m from the Clovis component of the Shawnee-Minisink site. (PDF)

S12 Table Counts of artifacts and bone 5 cm level from N 90 to 95 m and E 98 and 99 m from the Swan Point site. Relative elevations are distances above and below a plane fit through all artifacts deeper than 1.18 m below datum.
(PDF)

S1 File R code used in the study including function for calculation of ASI and simulation code. (TXT)

S2 File Simulation exploring sample size effects on ASI, site descriptions, and supplementary references. (DOCX)
