# Correction: Late date of human arrival to North America: Continental scale differences in stratigraphic integrity of pre-13,000 BP archaeological sites

**DOI:** 10.1371/journal.pone.0307979

**Published:** 2024-07-24

**Authors:** Todd A. Surovell, Sarah A. Allaun, Barbara A. Crass, Joseph A. M. Gingerich, Kelly E. Graf, Charles E. Holmes, Robert L. Kelly, Marcel Kornfeld, Kathryn E. Krasinski, Mary Lou Larson, Spencer R. Pelton, Brian T. Wygal

The Equation 2 is written incorrectly. Please view the complete, correct equation here:

$$ASI=\frac{\frac{\sum_{i=1}^{n-1}|f_{i}-f_{i+1}|}{n}}{2\frac{\sum_{i=1}^{n}f_{i}}{n}}$$
