# Supplementary figures and images for: Late date of human arrival to North America: Continental scale differences in stratigraphic integrity of pre-13,000 BP archaeological sites
Source: PLoS One. 2022 Apr 20;17(4):e0264092. doi: 10.1371/journal.pone.0264092 (PMC9020715; doi:10.1371/journal.pone.0264092)

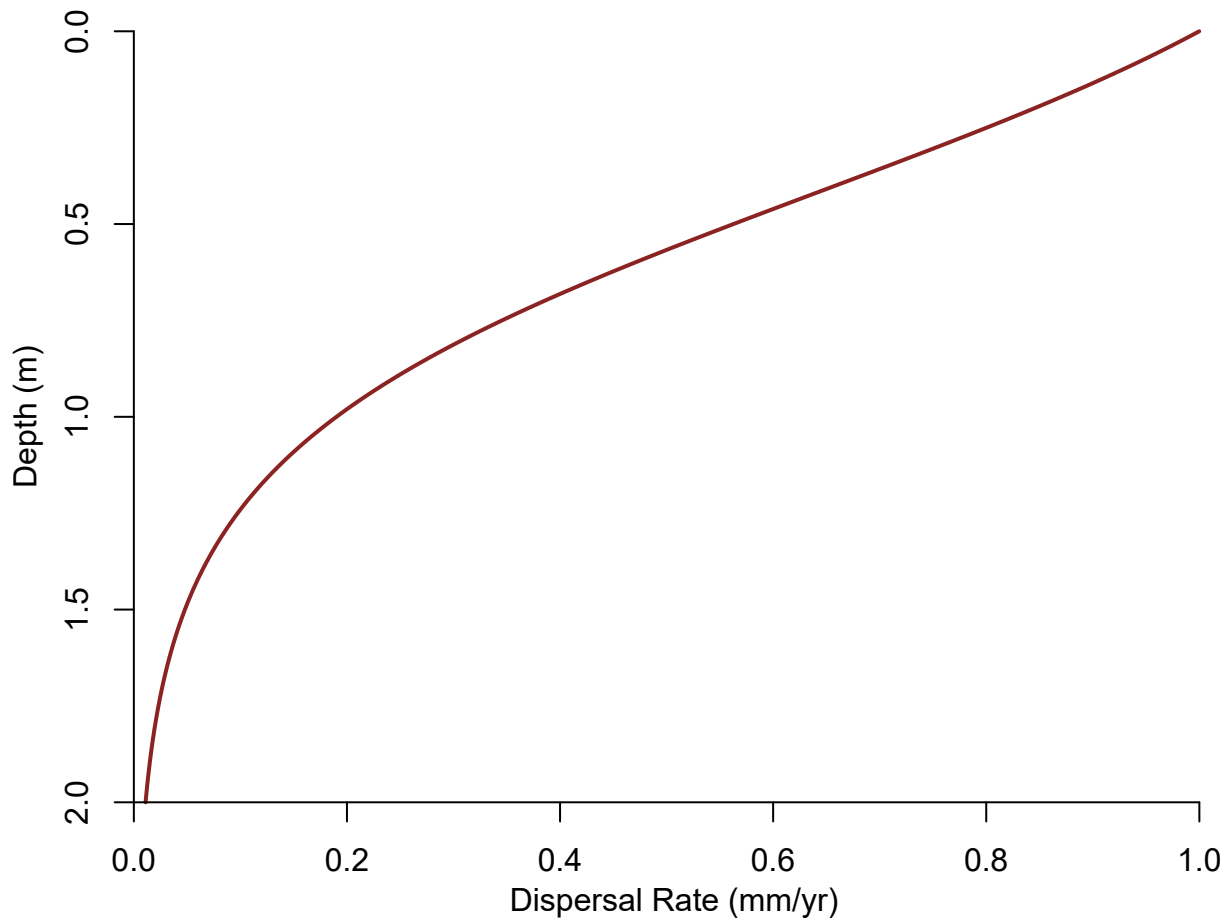

Supplement: S1 Fig — Shown for rmax = 1 mm/yr. (PDF) [file pone.0264092.s001.pdf]

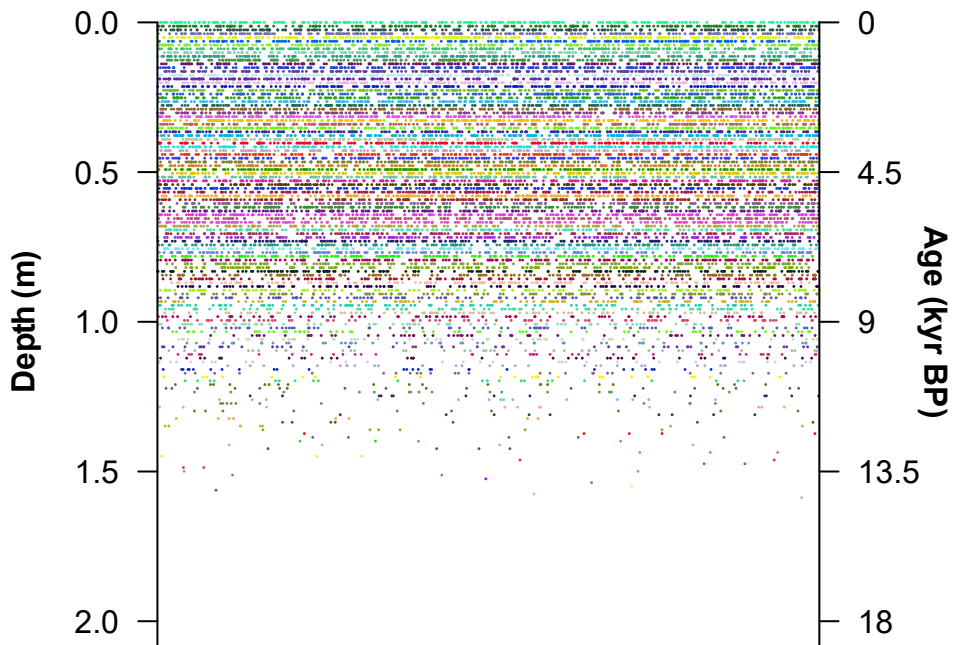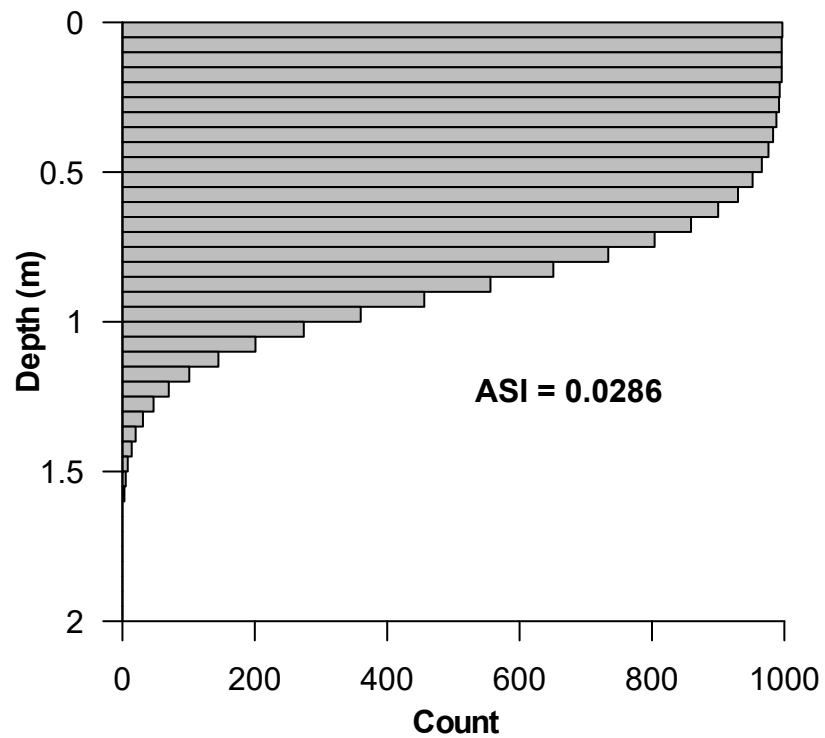

Supplement: S2 Fig — In this model, occupation intensity gradually increases over time and no vertical mixing occurs. In the backplot, artifacts are colored by occupation. (PDF) [file pone.0264092.s002.pdf]

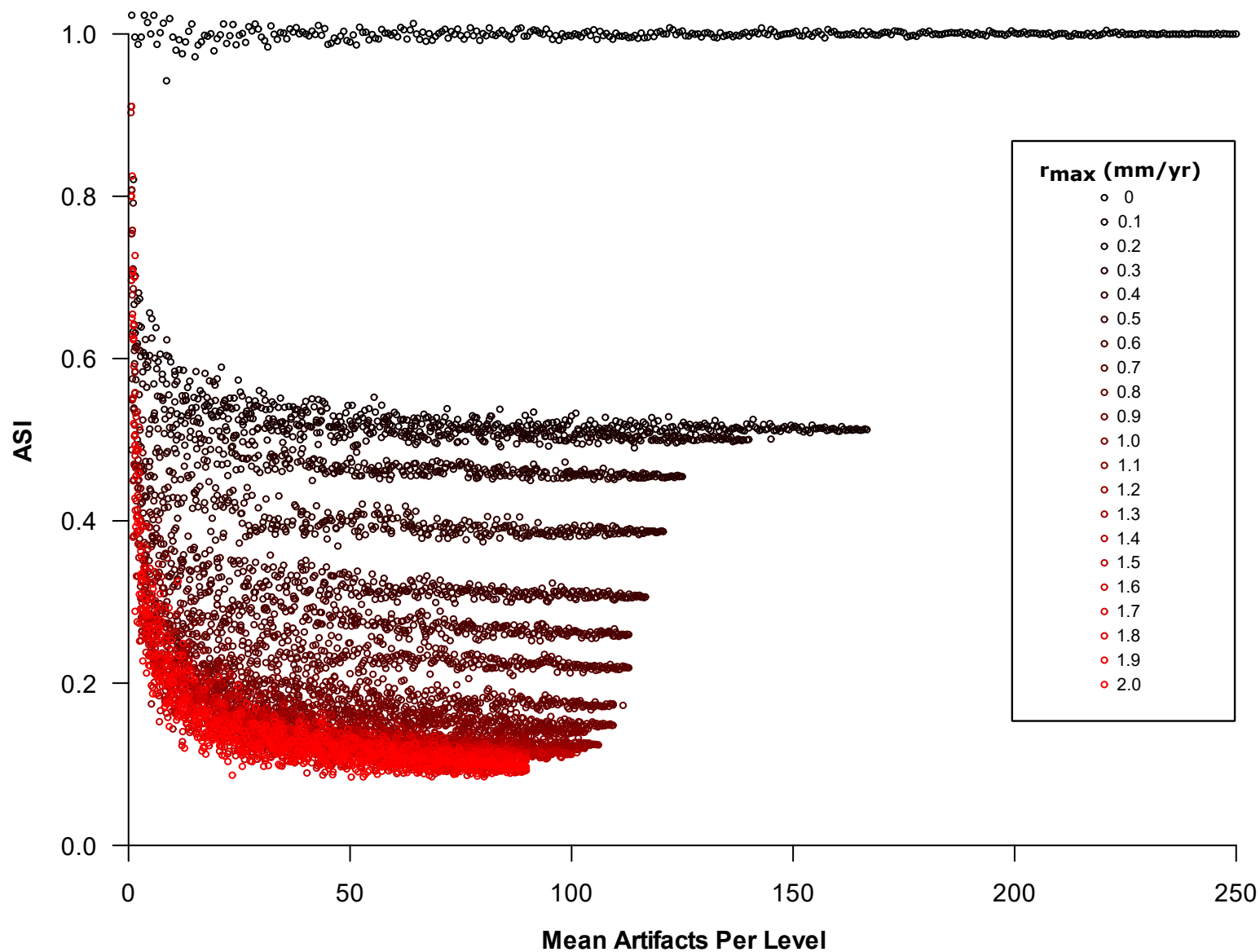

Supplement: S3 Fig — (PDF) [file pone.0264092.s003.pdf]

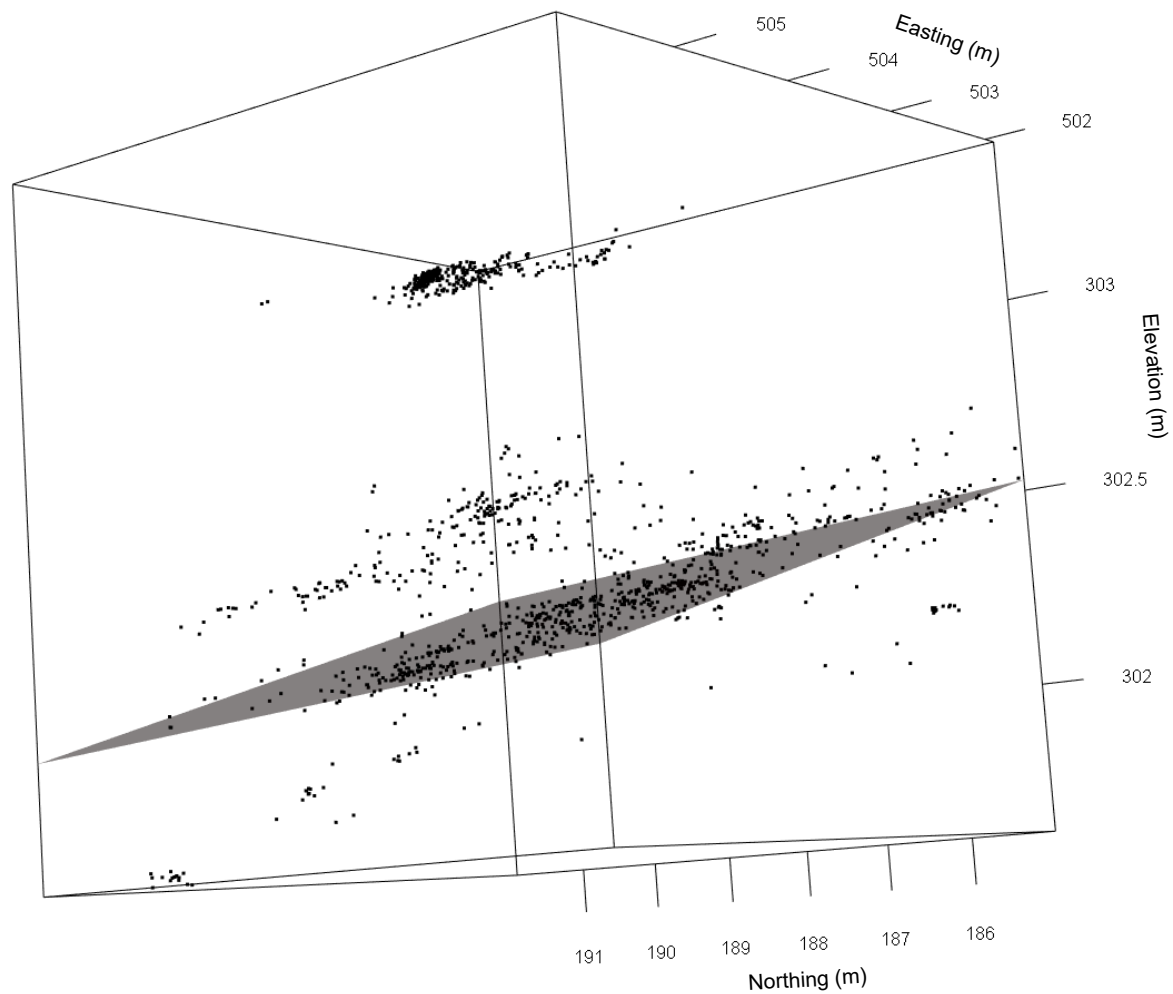

Supplement: S4 Fig — Three-dimensional scatterplot of artifacts from Holzman South with a plane fit by multiple linear regression to component 5a. (PDF) [file pone.0264092.s004.pdf]

## Hell Gap

ASI = 0.367

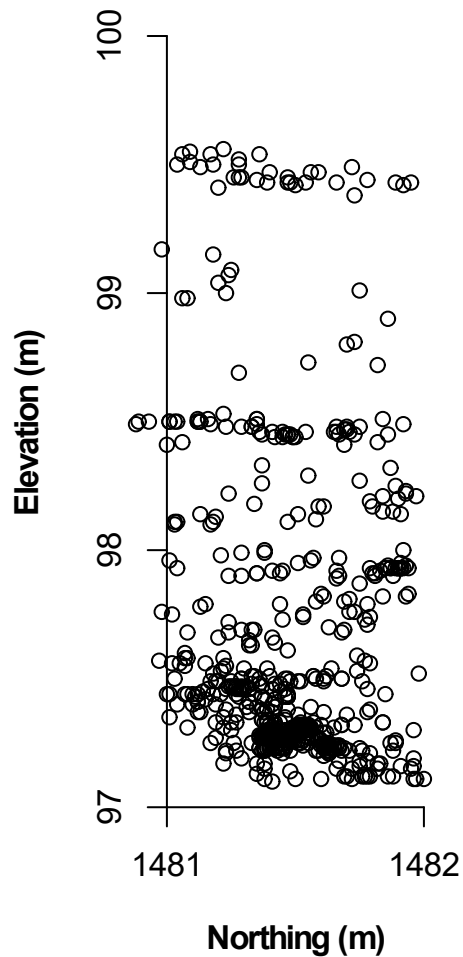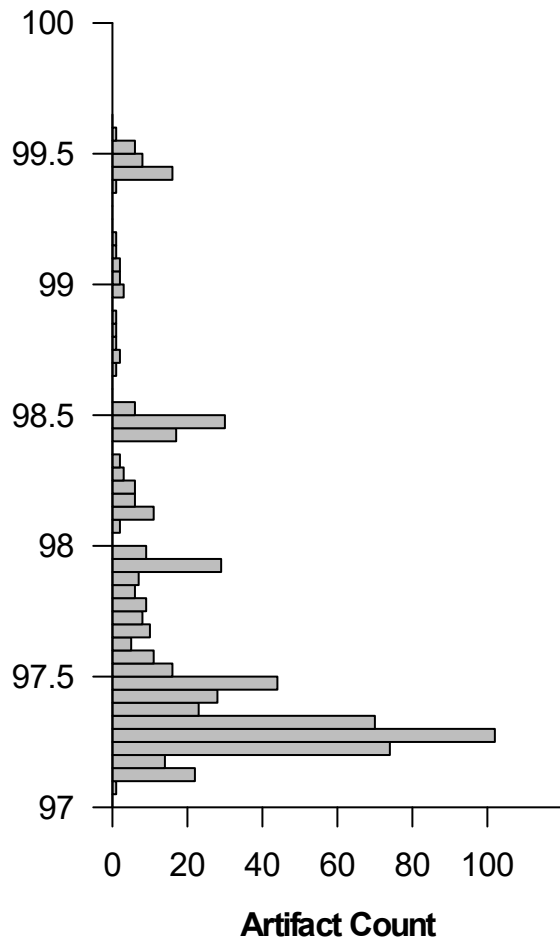

Supplement: S5 Fig — (PDF) [file pone.0264092.s005.pdf]

**Alm Shelter**

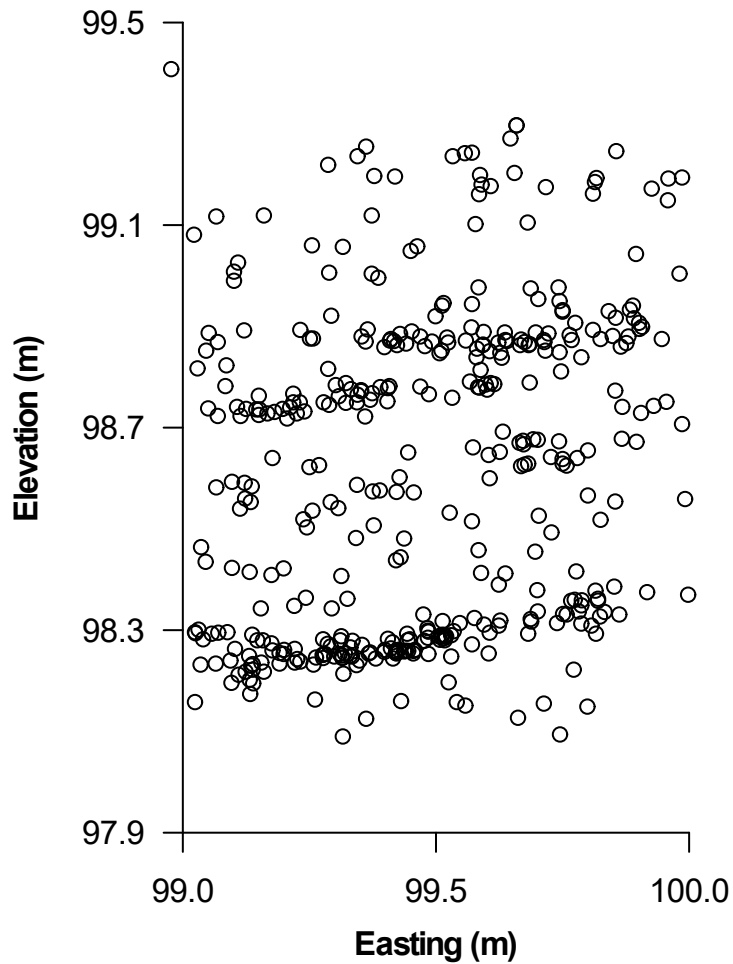

**ASI = 0.386**

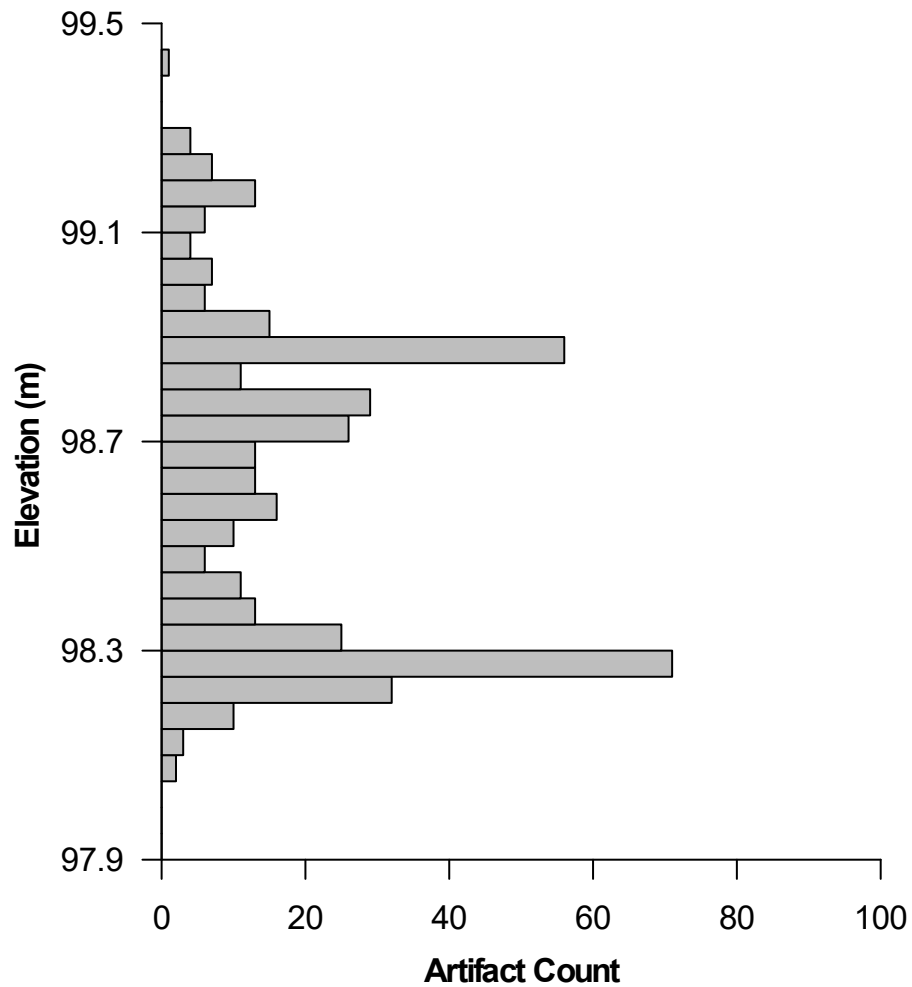

Supplement: S6 Fig — (PDF) [file pone.0264092.s006.pdf]

**Helen Lookingbill**

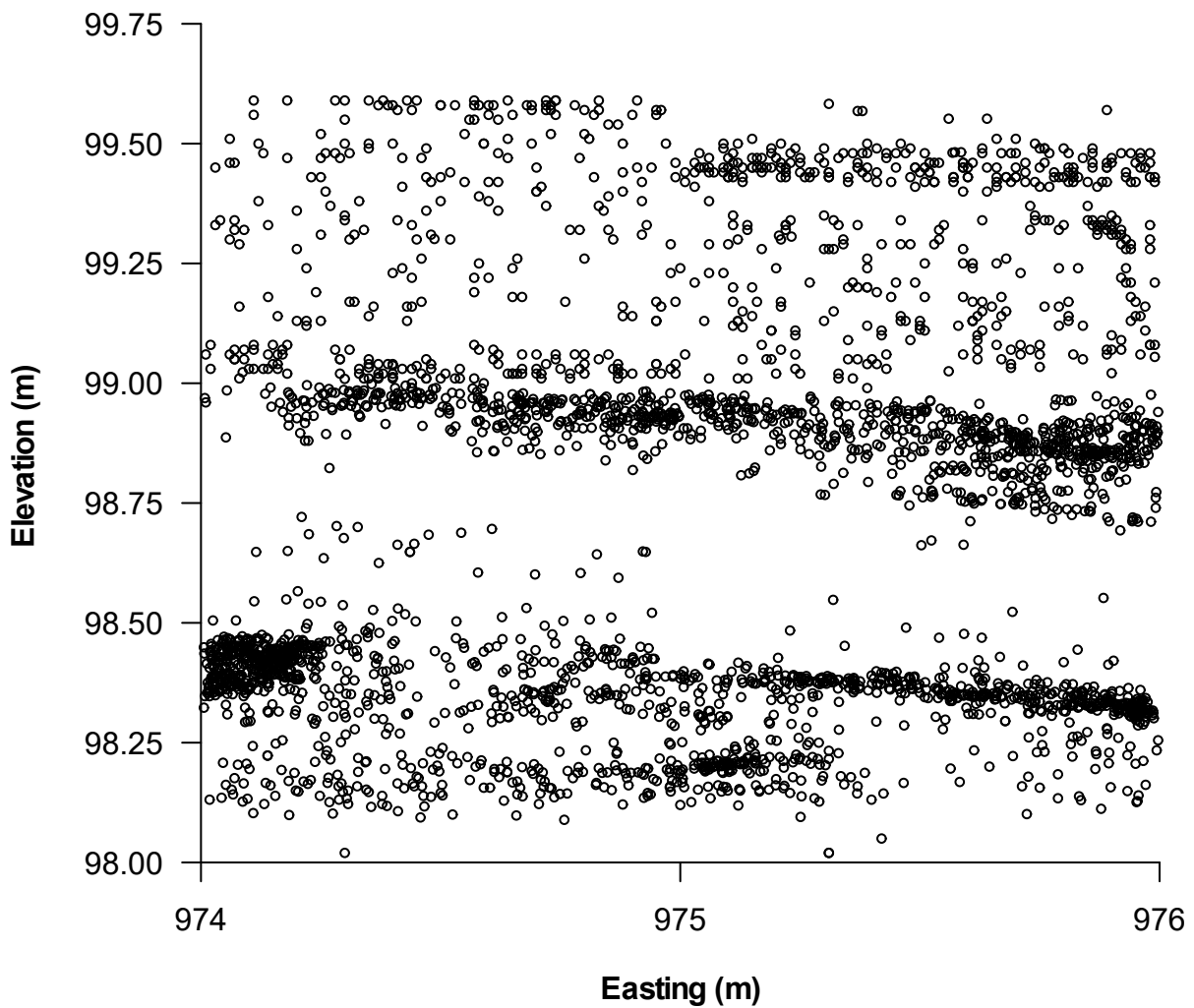

**ASI = 0.361**

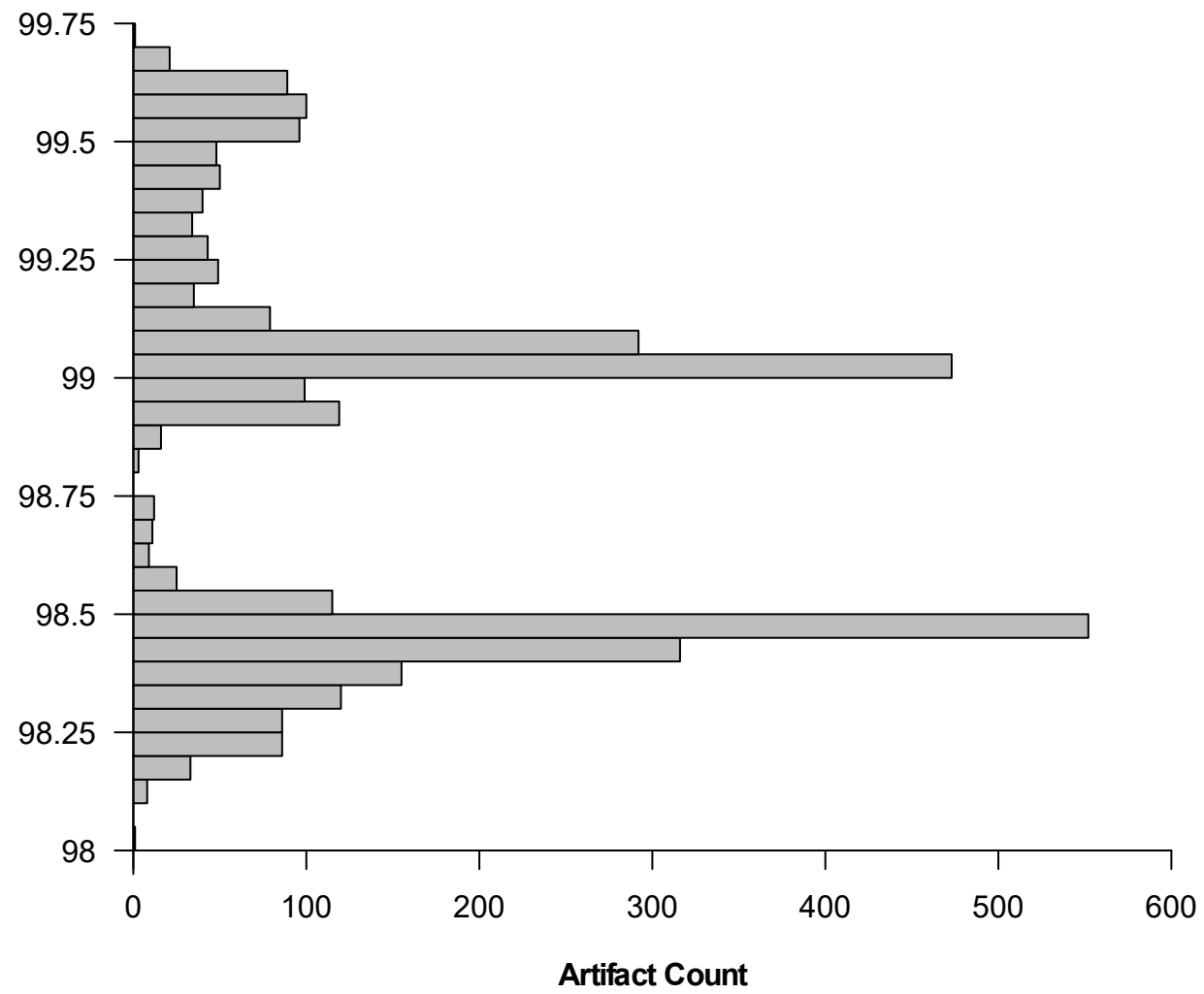

Supplement: S7 Fig — (PDF) [file pone.0264092.s007.pdf]

## Shawnee Minisink

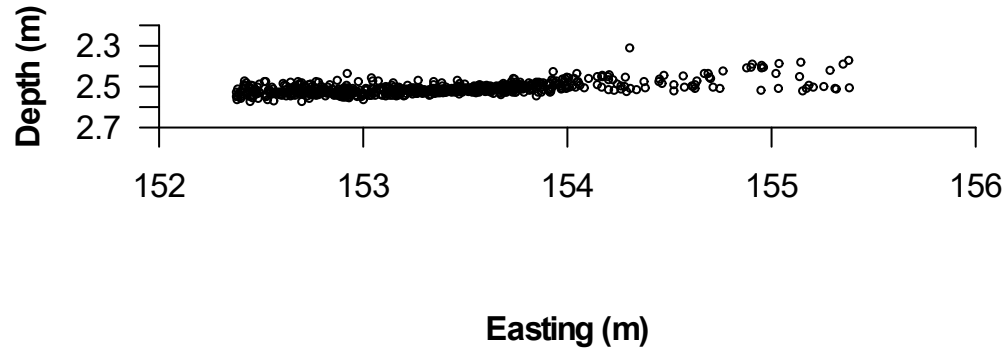

ASI = 0.676

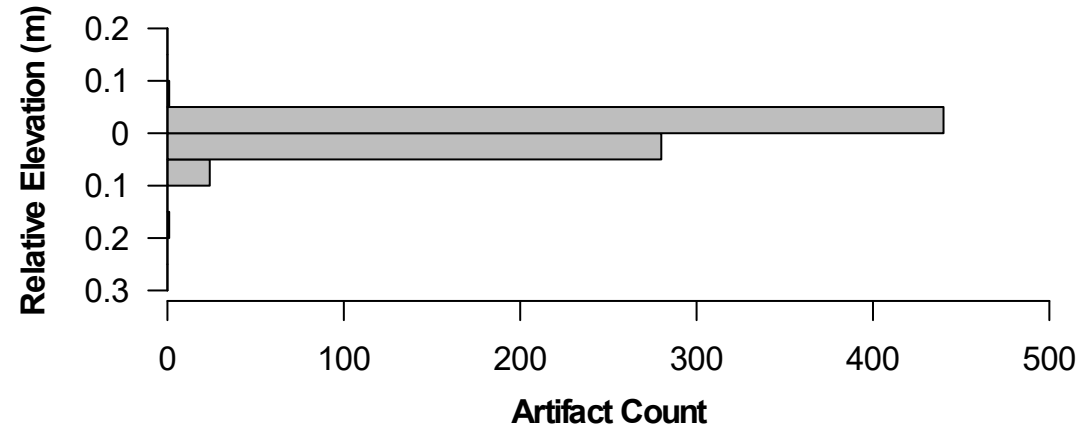

Supplement: S8 Fig — (PDF) [file pone.0264092.s008.pdf]

**ASI = 0.187**

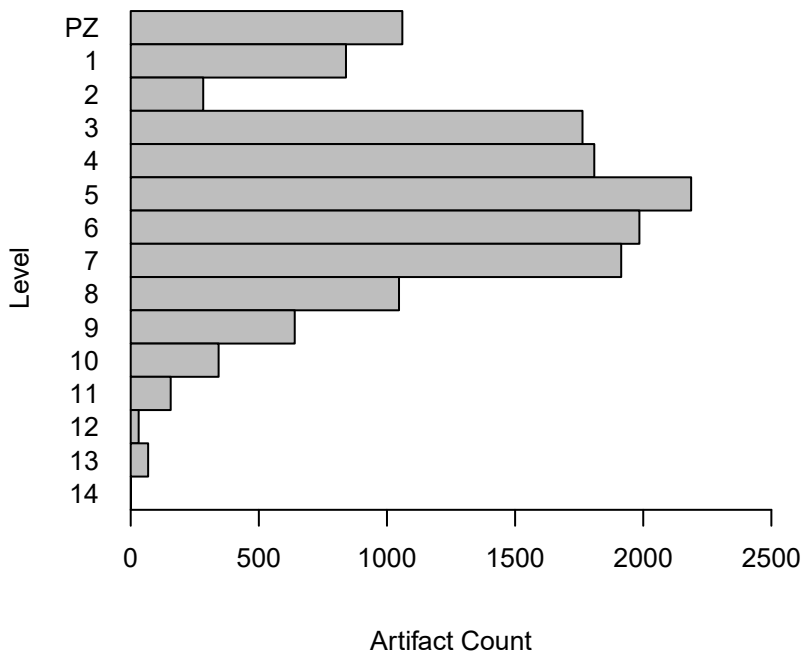

**ASI = 0.156**

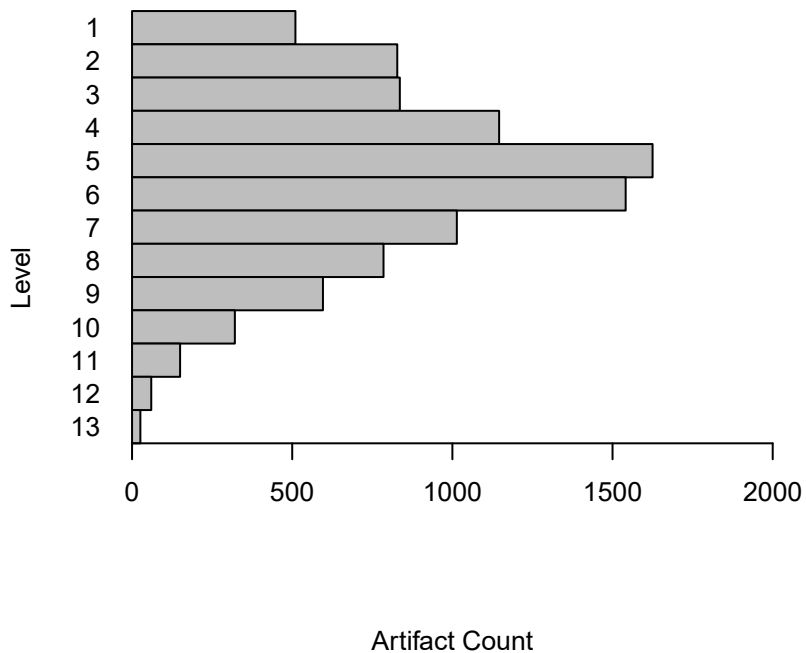

**ASI = 0.309**

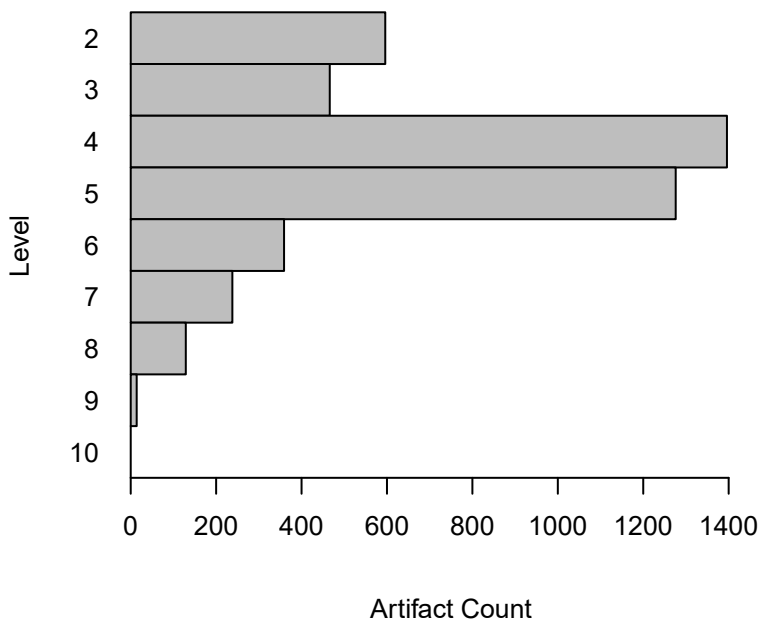

**ASI = 0.286**

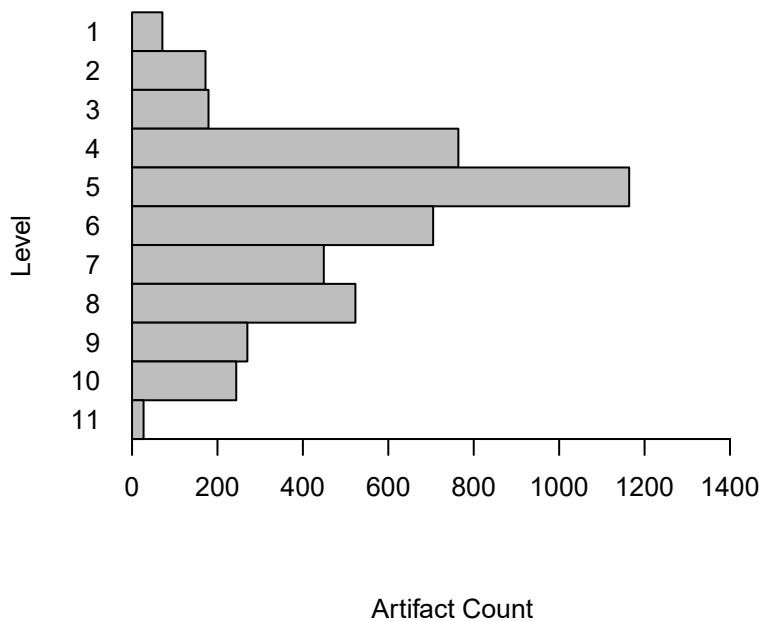

Supplement: S9 Fig — a. Block B, Unit 16; b. Block B, Unit 17; c. W165 N100; d. W115 N70. Excavation levels at Cactus Hill were not dug in uniform thicknesses. (PDF) [file pone.0264092.s009.pdf]
