# Supplementary material for: Late date of human arrival to North America: Continental scale differences in stratigraphic integrity of pre-13,000 BP archaeological sites
Source: PLoS One. 2022 Apr 20;17(4):e0264092. doi: 10.1371/journal.pone.0264092 (PMC9020715; doi:10.1371/journal.pone.0264092)
Supplement: S1 Table — (PDF) [file pone.0264092.s010.pdf]

Min Elev (m)	Max Elev (m)	Artifact Count
99.4	99.45	1
99.35	99.4	0
99.3	99.35	0
99.25	99.3	4
99.2	99.25	7
99.15	99.2	13
99.1	99.15	6
99.05	99.1	4
99	99.05	7
98.95	99	6
98.9	98.95	15
98.85	98.9	56
98.8	98.85	11
98.75	98.8	29
98.7	98.75	26
98.65	98.7	13
98.6	98.65	13
98.55	98.6	16
98.5	98.55	10
98.45	98.5	6
98.4	98.45	11
98.35	98.4	13
98.3	98.35	25
98.25	98.3	71
98.2	98.25	32
98.15	98.2	10
98.1	98.15	3
98.05	98.1	2
98	98.05	0

Table S1. Chipped stone artifact counts by 5 cm level for unit N98 E99 of Alm Shelter.
