# Supplementary material for: Late date of human arrival to North America: Continental scale differences in stratigraphic integrity of pre-13,000 BP archaeological sites
Source: PLoS One. 2022 Apr 20;17(4):e0264092. doi: 10.1371/journal.pone.0264092 (PMC9020715; doi:10.1371/journal.pone.0264092)
Supplement: S2 Table — Relative elevation in the distance above or below a plane fit through the LP component. (PDF) [file pone.0264092.s011.pdf]

Min Rel. Elev. (m)	Max. Rel. Elev. (m)	Count
1.3	1.35	1
1.25	1.3	7
1.2	1.25	26
1.15	1.2	39
1.1	1.15	12
1.05	1.1	3
1	1.05	0
0.95	1	2
0.9	0.95	0
0.85	0.9	1
0.8	0.85	4
0.75	0.8	0
0.7	0.75	0
0.65	0.7	0
0.6	0.65	0
0.55	0.6	0
0.5	0.55	0
0.45	0.5	0
0.4	0.45	0
0.35	0.4	0
0.3	0.35	2
0.25	0.3	9
0.2	0.25	34
0.15	0.2	38
0.1	0.15	33
0.05	0.1	68
0	0.05	206
-0.05	0	172
-0.1	-0.05	43
-0.15	-0.1	18
-0.2	-0.15	3
-0.25	-0.2	2
-0.3	-0.25	1

Table S2. Artifact and bone counts by 5 cm level for N 96 to 100 m and E 110 to 199 m of the Broken Mammoth site. Relative elevation in the distance above or below a plane fit through the LP component.
