# Supplementary material for: Late date of human arrival to North America: Continental scale differences in stratigraphic integrity of pre-13,000 BP archaeological sites
Source: PLoS One. 2022 Apr 20;17(4):e0264092. doi: 10.1371/journal.pone.0264092 (PMC9020715; doi:10.1371/journal.pone.0264092)
Supplement: S3 Table — (PDF) [file pone.0264092.s012.pdf]

Min Elev (masl)	Max Elev. (masl)	Count
411.85	411.9	35
411.8	411.85	27
411.75	411.8	31
411.7	411.75	29
411.65	411.7	21
411.6	411.65	36
411.55	411.6	14
411.5	411.55	14
411.45	411.5	17
411.4	411.45	7
411.35	411.4	3
411.3	411.35	6
411.25	411.3	4
411.2	411.25	6
411.15	411.2	2
411.1	411.15	1
411.05	411.1	1
411	411.05	0

Table S3. Artifact and bone counts by 5 cm level for stratum LU3 of Area A of the Cooper's Ferry site.
