# Supplementary material for: Late date of human arrival to North America: Continental scale differences in stratigraphic integrity of pre-13,000 BP archaeological sites
Source: PLoS One. 2022 Apr 20;17(4):e0264092. doi: 10.1371/journal.pone.0264092 (PMC9020715; doi:10.1371/journal.pone.0264092)
Supplement: S4 Table — (PDF) [file pone.0264092.s013.pdf]

Min Elev. (m)	Max Elev. (m)	Artifact Count
91.1	91.15	14070
91.05	91.1	12290
91	91.05	11970
90.95	91	11510
90.9	90.95	10770
90.85	90.9	10620
90.8	90.85	10730
90.75	90.8	10600
90.7	90.75	9710
90.65	90.7	9320
90.6	90.65	9210
90.55	90.6	8440
90.5	90.55	7250
90.45	90.5	6470
90.4	90.45	5580
90.35	90.4	4450
90.3	90.35	3870
90.25	90.3	2630
90.2	90.25	2010
90.15	90.2	1270
90.1	90.15	680
90.05	90.1	300
90	90.05	170
89.95	90	0

Table S4. Debitage and tool count by 5 cm level for Block A of the Debra Friedkin site.
