# Supplementary material for: Late date of human arrival to North America: Continental scale differences in stratigraphic integrity of pre-13,000 BP archaeological sites
Source: PLoS One. 2022 Apr 20;17(4):e0264092. doi: 10.1371/journal.pone.0264092 (PMC9020715; doi:10.1371/journal.pone.0264092)
Supplement: S5 Table — Relative elevation in the distance above or below a plane fit through Component 2. (PDF) [file pone.0264092.s014.pdf]

Min Rel. Elev. (m)	Max Rel. Elev. (m)	Artifact Count
0.1	0.15	4
0.05	0.1	102
0	0.05	509
-0.05	0	547
-0.1	-0.05	21
-0.15	-0.1	4
-0.2	-0.15	4
-0.25	-0.2	16
-0.3	-0.25	8
-0.35	-0.3	14
-0.4	-0.35	15
-0.45	-0.4	0
-0.5	-0.45	0
-0.55	-0.5	1
-0.6	-0.55	0

Table S5. Chipped stone artifact counts by 5 cm level for N 14 to 16 m and E 21 to 23 m of the Dry Creek site. Relative elevation in the distance above or below a plane fit through Component 2.
