# Supplementary material for: Late date of human arrival to North America: Continental scale differences in stratigraphic integrity of pre-13,000 BP archaeological sites
Source: PLoS One. 2022 Apr 20;17(4):e0264092. doi: 10.1371/journal.pone.0264092 (PMC9020715; doi:10.1371/journal.pone.0264092)
Supplement: S6 Table — (PDF) [file pone.0264092.s015.pdf]

Min Elev. (m)	Max Elev. (m)	Flake Count
94.3	94.35	1590
94.25	94.3	700
94.2	94.25	630
94.15	94.2	600
94.1	94.15	510
94.05	94.1	550
94	94.05	600
93.95	94	370
93.9	93.95	430
93.85	93.9	450
93.8	93.85	370
93.75	93.8	280
93.7	93.75	470
93.65	93.7	510
93.6	93.65	470
93.55	93.6	630
93.5	93.55	500
93.45	93.5	1030
93.4	93.45	660
93.35	93.4	650
93.3	93.35	710
93.25	93.3	500
93.2	93.25	220
93.15	93.2	250
93.1	93.15	300
93.05	93.1	250
93	93.05	860
92.95	93	560
92.9	92.95	610
92.85	92.9	470
92.8	92.85	330
92.75	92.8	330
92.7	92.75	320
92.65	92.7	1150
92.6	92.65	1000
92.55	92.6	910
92.5	92.55	530
92.45	92.5	550
92.4	92.45	560
92.35	92.4	530
92.3	92.35	590
92.25	92.3	650
92.2	92.25	550
92.15	92.2	1580
92.1	92.15	1560
92.05	92.1	4290
92	92.05	1640
91.95	92	910
91.9	91.95	180
91.85	91.9	0

Table S6. Counts of flakes by 5 cm level from Area 15 of the Gault site.
