# Supplementary material for: Late date of human arrival to North America: Continental scale differences in stratigraphic integrity of pre-13,000 BP archaeological sites
Source: PLoS One. 2022 Apr 20;17(4):e0264092. doi: 10.1371/journal.pone.0264092 (PMC9020715; doi:10.1371/journal.pone.0264092)
Supplement: S7 Table — Relative elevations are the distance above or below a plane fit through all artifacts between elevations 98.7 and 99.1 m. (PDF) [file pone.0264092.s016.pdf]

Min Rel. Elev. (m)	Max Rel. Elev. (m)	Count
0.7	0.75	1
0.65	0.7	1
0.6	0.65	34
0.55	0.6	83
0.5	0.55	87
0.45	0.5	52
0.4	0.45	47
0.35	0.4	47
0.3	0.35	28
0.25	0.3	33
0.2	0.25	39
0.15	0.2	40
0.1	0.15	26
0.05	0.1	102
0	0.05	406
-0.05	0	293
-0.1	-0.05	101
-0.15	-0.1	72
-0.2	-0.15	3
-0.25	-0.2	0
-0.3	-0.25	0
-0.35	-0.3	2
-0.4	-0.35	1
-0.45	-0.4	1
-0.5	-0.45	10
-0.55	-0.5	320
-0.6	-0.55	243
-0.65	-0.6	315
-0.7	-0.65	168
-0.75	-0.7	40
-0.8	-0.75	11
-0.85	-0.8	9
-0.9	-0.85	6
-0.95	-0.9	0
-1	-0.95	0
-1.05	-1	1
-1.1	-1.05	0

Table S7. Chipped stone artifact and bone counts by 5 cm level for N 1014 to 1015 m and E 974 to 976 m from the Helen Lookingbill site. Relative elevations are the distance above or below a plane fit through all artifacts between elevations 98.7 and 99.1 m.
