# Supplementary material for: Late date of human arrival to North America: Continental scale differences in stratigraphic integrity of pre-13,000 BP archaeological sites
Source: PLoS One. 2022 Apr 20;17(4):e0264092. doi: 10.1371/journal.pone.0264092 (PMC9020715; doi:10.1371/journal.pone.0264092)
Supplement: S8 Table — (PDF) [file pone.0264092.s017.pdf]

Min. Elev. (m)	Max. Elev. (m)	Count
99.6	99.65	0
99.55	99.6	1
99.5	99.55	6
99.45	99.5	8
99.4	99.45	16
99.35	99.4	1
99.3	99.35	0
99.25	99.3	0
99.2	99.25	0
99.15	99.2	1
99.1	99.15	1
99.05	99.1	2
99	99.05	2
98.95	99	3
98.9	98.95	0
98.85	98.9	1
98.8	98.85	1
98.75	98.8	1
98.7	98.75	2
98.65	98.7	1
98.6	98.65	0
98.55	98.6	0
98.5	98.55	6
98.45	98.5	30
98.4	98.45	17
98.35	98.4	0
98.3	98.35	2
98.25	98.3	3
98.2	98.25	6
98.15	98.2	6
98.1	98.15	11
98.05	98.1	2
98	98.05	0
97.95	98	9
97.9	97.95	29
97.85	97.9	7
97.8	97.85	6
97.75	97.8	9
97.7	97.75	8
97.65	97.7	10
97.6	97.65	5
97.55	97.6	11
97.5	97.55	16
97.45	97.5	44
97.4	97.45	28
97.35	97.4	23
97.3	97.35	70
97.25	97.3	102
97.2	97.25	74
97.15	97.2	14
97.1	97.15	22
97.05	97.1	1
97	97.05	0

Table S8. Counts of chipped stone artifacts, ocher, and bone by 5 cm level from N 1481 to 1482 m and E 1294.5 and 1296.1 m of Locality I of the Hell Gap site.
