# Supplementary material for: Late date of human arrival to North America: Continental scale differences in stratigraphic integrity of pre-13,000 BP archaeological sites
Source: PLoS One. 2022 Apr 20;17(4):e0264092. doi: 10.1371/journal.pone.0264092 (PMC9020715; doi:10.1371/journal.pone.0264092)
Supplement: S9 Table — Relative elevations are the distance above or below a plane fit through artifacts from Component 5a. (PDF) [file pone.0264092.s018.pdf]

Min Rel. Elev. (m)	Max Rel. Elev. (m)	Count
1	1.05	3
0.95	1	354
0.9	0.95	419
0.85	0.9	19
0.8	0.85	0
0.75	0.8	0
0.7	0.75	0
0.65	0.7	0
0.6	0.65	0
0.55	0.6	0
0.5	0.55	0
0.45	0.5	1
0.4	0.45	8
0.35	0.4	3
0.3	0.35	32
0.25	0.3	59
0.2	0.25	77
0.15	0.2	50
0.1	0.15	18
0.05	0.1	45
0	0.05	341
-0.05	0	354
-0.1	-0.05	40
-0.15	-0.1	4
-0.2	-0.15	1
-0.25	-0.2	11
-0.3	-0.25	27
-0.35	-0.3	14
-0.4	-0.35	5
-0.45	-0.4	0

Table S9. Artifact and bone counts by 5 cm level for N 185 to 192 m and E 502 to 506 m from the Holzman South site. Relative elevations are the distance above or below a plane fit through artifacts from Component 5a.
