# Supplementary material for: Late date of human arrival to North America: Continental scale differences in stratigraphic integrity of pre-13,000 BP archaeological sites
Source: PLoS One. 2022 Apr 20;17(4):e0264092. doi: 10.1371/journal.pone.0264092 (PMC9020715; doi:10.1371/journal.pone.0264092)
Supplement: S10 Table — (PDF) [file pone.0264092.s019.pdf]

Min Elev. (m)	Max Elev. (m)	Count
99.45	99.5	1
99.4	99.45	0
99.35	99.4	1
99.3	99.35	1
99.25	99.3	0
99.2	99.25	1
99.15	99.2	1
99.1	99.15	5
99.05	99.1	1
99	99.05	31
98.95	99	31
98.9	98.95	0
98.85	98.9	1
98.8	98.85	0

Table S10. Artifact counts by 5 cm level for N 87 to 89 m and E 110 to 111 m from the Owl Ridge site.
