# Supplementary material for: Late date of human arrival to North America: Continental scale differences in stratigraphic integrity of pre-13,000 BP archaeological sites
Source: PLoS One. 2022 Apr 20;17(4):e0264092. doi: 10.1371/journal.pone.0264092 (PMC9020715; doi:10.1371/journal.pone.0264092)
Supplement: S11 Table — (PDF) [file pone.0264092.s020.pdf]

Min Rel. Elev. (m)	Max Rel. Elev. (m)	Artifact Count
-0.2	-0.15	1
-0.15	-0.1	0
-0.1	-0.05	24
-0.05	0	280
0	0.05	440
0.05	0.1	1
0.1	0.15	0

Table S11. Artifact counts by 5 cm level for N 158.9 to 158.4 m and E 152.4 to 155.4 m from the Clovis component of the Shawnee-Minisink site.
