# Supplementary material for: Late date of human arrival to North America: Continental scale differences in stratigraphic integrity of pre-13,000 BP archaeological sites
Source: PLoS One. 2022 Apr 20;17(4):e0264092. doi: 10.1371/journal.pone.0264092 (PMC9020715; doi:10.1371/journal.pone.0264092)
Supplement: S12 Table — Relative elevations are distances above and below a plane fit through all artifacts deeper than 1.18 m below datum. (PDF) [file pone.0264092.s021.pdf]

Min. Rel. Elev (m)	Max. Rel. Elev. (m)	Count
0.8	0.85	2
0.75	0.8	9
0.7	0.75	9
0.65	0.7	15
0.6	0.65	24
0.55	0.6	49
0.5	0.55	37
0.45	0.5	12
0.4	0.45	3
0.35	0.4	20
0.3	0.35	8
0.25	0.3	1
0.2	0.25	0
0.15	0.2	1
0.1	0.15	3
0.05	0.1	27
0	0.05	453
-0.05	0	570
-0.1	-0.05	55
-0.15	-0.1	5
-0.2	-0.15	2
-0.25	-0.2	0

Table S12. Counts of artifacts and bone 5 cm level from N 90 to 95 m and E 98 and 99 m from the Swan Point site. Relative elevations are distances above and below a plane fit through all artifacts deeper than 1.18 m below datum.
