# Supplementary material for: Late date of human arrival to North America: Continental scale differences in stratigraphic integrity of pre-13,000 BP archaeological sites
Source: PLoS One. 2022 Apr 20;17(4):e0264092. doi: 10.1371/journal.pone.0264092 (PMC9020715; doi:10.1371/journal.pone.0264092)
Supplement: S2 File — (DOCX) [file pone.0264092.s023.docx]

**Sample Size vs ASI Simulation**

We created a simulation to explore the relationship between assemblage size and the ASI. We varied the maximum dispersal rate (*r_max_*) from 0 to 2 mm per year in increments of 0.1 mm in the standard simulation in which sediments accumulate at a constant rate of 0.1 mm per year over 18,000 years and seven occupations occur spaced 2,000 years apart from 12,500 to 500 BP. For each occupation, 500 artifacts are discarded resulting in a total assemblage size of 3,500 pieces. To vary sample size, we then randomly sampled 10 to 3,500 artifacts from each simulation and calculated the resulting ASI. Results are shown in Figure S2. With no mixing at all *(r_max_* = 0 mm/yr), ASI is unaffected by sample size. Once mixing is added to the system, however, ASI values are impacted by small sample sizes because as artifact counts drop, vertical density distributions increasingly show gaps thus inflating ASI values. ASI values stabilize as assemblage size increases. In general, ASIs are expected to be inflated when there are fewer than a mean of ~30 artifacts per level, and they can be very inaccurate and inflated for sites with fewer than 10 artifacts per level. With fewer than 10 artifacts per level, it becomes difficult to distinguish between intact and highly mixed archaeological deposits using vertical artifact distributions.

**Why ASI Alone Cannot Establish Stratigraphic Integrity**

To illustrate why the ASI alone cannot establish stratigraphic integrity, we created a simulated case that couples frequent reoccupation with gradually increasing occupation intensity and a low rate of deposition. In this simulation, we use our standard depositional model in which sediments accumulate at a constant rate of 0.1 mm per year over 18,000 years. Occupations (n=128) are spaced 126 years apart with the first occupation occurring at 16,002 BP. The number of artifacts discarded increases logistically from one to 249. We set *r_max_* to zero, so no vertical mixing affects artifact distributions. The results are shown in Figure S2. Although this site has no mixing whatsoever, its vertical distribution produces a very low ASI value of 0.029. While the existence of such a site is theoretically possible, it would require the unlikely perfect convergence of events in which occupation intensity gradually increases over time with no variation from that trend coupled with absolutely no disturbance. If this were to occur, a vertical distribution would result that looks nearly identical to one generated by significant vertical disturbance and no change in occupation intensity. To distinguish between these two possibilities would require the use of independent lines of evidence and highlights why studies of artifact inclination, size sorting, and especially refitting are critical to establishing site integrity.

It is also worth pointing out that sites with high ASI values and low stratigraphic integrity are also possible. A reoccupied site with significant bioturbation such that biomantles or stone lines are formed [1,2] could theoretically generate a high ASI value, as could a multicomponent site characterized by buried deflation surfaces or desert pavements. In a case like this, archaeological evidence would likely be insufficient to distinguish between the highly mixed and unmixed scenarios, except perhaps if diagnostic artifacts of dramatically different ages were stratigraphically comingled. Geomorphic evidence, however, could likely be used if, for example, natural and cultural clasts showed similar stratigraphic distributions [3].

**Mathematical and Statistical Properties of the ASI**

Though we lack proof, we suspect the maximum possible value of the ASI is 1.5. This result is attained when *n*=3 and the central value is a nonzero positive integer bracketed by zeroes. If we are correct that the maximum value is 1.5, the ASI could be standardized to vary from zero to one using a coefficient of 3, instead of 2, in the denominator. We hesitate to make this change in its calculation first because it would compress the variability in the observed archaeological cases, and as currently formulated, we feel it captures real world variability well. Nonetheless, if the change was made, relative ASI differences for each site in this study would remain unchanged, and our conclusions would remain the same. Furthermore, it is easily shown that for all cases in which *n* is even and the vector is comprised of alternating values of zero and a constant integer *x*, the ASI is equal to 1.0. If the denominator coefficient was changed to 3, these cases would yield an ASI of 2/3, and we suggest that a value of 1.0 is more useful as an intuitive result. With our formulation of ASI, vectors comprised of zeroes alternating with a constant integer and with zeroes as the starting and ending values, decline from 1.5 to 1.0 as sample size *n* increases. The ASI also increases from 0.75 to a limiting value of 1.0 as sample size increases for vectors of alternating zeroes and constant integers if the nonzero integer value begins and ends the vector.

We also explored how ASI values are distributed for simulated cases. We created sampling distributions of several simulated cases using different levels of *r_max_* and sample sizes. For those cases, sampling distributions of the ASI seemed to be best described by the gamma distribution, though this problem would be better explored using actual rather than simulated data. We consider our formulation of ASI to be a first attempt at measuring relative mean absolute successive difference and look forward to future investigators using, modifying, and improving this statistic.

**Site Descriptions**

*Alm Shelter, Wyoming*

Alm Shelter lies at the base of a limestone cliff on the west side of the Bighorn Mountains near the mouth of Paint Rock Canyon[4,5]. Though excavation (2005, 2009, 2014-18) penetrated to sediments dating to >18,000 BP, the earliest occupation is ~12,000 BP. Based on 26 radiocarbon dates and hundreds of piece-plots, the primary components are Late Paleoindian, Early Archaic, and Middle Archaic; use of the site declined during the Late Archaic and was rare in the Late Prehistoric period. Maximum depth of the excavation was 360 cm below surface (stream gravels), with most archaeological remains lying 40-200 cm below surface. The lack of visual evidence of bioturbation; refitted artifacts; thin, intact loess layers (prior to 6400 BP); and the stratigraphic order of the ^14^C dates (only two reversals, one minor) all point to a very intact stratigraphy.

Vertical density data were based on all piece-plotted artifacts from the square meter unit N98 E99. Artifacts span more than 1.3 m of depth. No correction was applied for stratigraphic tilt. The ASI value for Alm could be somewhat inflated due to low artifact counts. For this part of the site, there is a mean of 14.6 piece-plotted artifacts per 5 cm level.

*Broken Mammoth, Alaska*

Broken Mammoth is a multicomponent site spanning the Holocene and Late Pleistocene and overlooks the Tanana River near the confluence of Shaw Creek in central Alaska. Holmes and Yesner began excavations in 1990 [6,7]. Four main components occur in over 2 m of stratified loess with the oldest, component 4, dating between ca. 12,900 and 13,600 BP (nine radiocarbon dates on charcoal and collagen). Component 4 is confined to the lower paleosol complex “…2-3 continuous to discontinuous, dark grayish brown (10YR 4/2), undulating, organic layers each up to 4 cm thick ….” [8], at between 1.5 and 1.6 m depth; and contains both lithic and bone/tusk artifacts associated with a variety of bird and mammal remains scattered around a series of discretely contained hearth features [9].

Vertical density data are based on all piece-plotted artifacts and bone between N 96 and 100 m and between E 110 and 119m. Elevations were adjusted by fitting a plane through the Late Pleistocene component using multiple linear regression. Artifact relative elevations are calculated as the distance above or below that plane. Artifacts span 2.17 m of depth. The ASI value for Broken Mammoth could be slightly inflated due to low artifact counts. For this part of the site, there is a mean of 29.0 piece-plotted artifacts per 5 cm level.

*Cooper’s Ferry, Idaho*

Cooper’s Ferry is a multicomponent site that occurs in an alluvial terrace where Rock Creek joins the Salmon River in Idaho County, Idaho. The site was first excavated by Butler in the 1960s [10] and later by Davis and colleagues [11–13]. Cultural components are preserved in over 2 m of sediments dating to the Late Pleistocene and Early Holocene. Multiple occupations associated with stemmed projectile points are present. The deepest cultural materials in the site occur in stratum LU3, and the first use of the site by humans is argued to date to around 16,000 BP based on several radiocarbon and OSL ages [12]**.** In addition to chipped stone artifacts and bone, pit and hearth features are argued to be present. There is ongoing debate as to what extent bioturbation has affected artifact distributions [14,15].

Artifact distribution data for this study were taken from Table S4 from Davis et al. 2019 [12]. These are from Area A of the site, excavated from 2009-2018. Our analysis includes all piece-plotted chipped stone artifacts and bone outside of cultural features from the deepest deposits in the site (LU3). Artifact counts by level from the upper parts of the site are not available. Because no discrete cultural levels and no stratigraphic tilt is evident in the backplot, we did not attempt to correct for sloping beds. Published profiles also suggest little if any slope is present [12]. To calculate an ASI, bone and chipped stone counts were binned in 5 cm levels using reported elevations. We repeated our analysis using only chipped stone, excluding bone, and found it to be virtually identical to the value if bone is included (bone and chipped stone, ASI = 0.186; chipped stone only, ASI=0.187). Using bone and stone, there is a mean of 14.1 artifacts per 5 cm level, so these ASI values could be somewhat inflated.

*Debra L. Friedkin, Texas*

The Debra L. Friedkin site is approximately 250 m to 400 m downstream of the Gault site in Bell County of central Texas [16–20] in a small tributary valley of the Brazos River called Buttermilk Creek. Cultural components are buried in 1.2 to 1.4 m of slowly aggrading overbank deposits overlying colluvium and limestone bedrock. Two areas of the site have been excavated; our analysis uses data from Block A. The site setting is similar to that of the Gault site (see below), although sedimentation rates were much lower resulting in a somewhat compressed cultural sequence compared to what is observed at Gault. The entire prehistoric sequence is present, but most of the deposits span the Paleoindian through Middle Archaic periods. Age control is provided by numerous luminescence dates, the oldest of which date to nearly 25,000 BP. The initial occupation of the site is believed to have occurred around 15,500 BP [19,20]. The site is argued to retain stratigraphic integrity based on geomorphic evidence [16,20,21] and minimal vertical mixing of diagnostic artifacts [19], but others have questioned the site’s integrity based on apparent artifact size sorting [22].

We did not have access to data on the locations of artifacts mapped *in situ*, so vertical artifact counts for 5 cm layers were obtained by digitization of Figure 4a from Waters et al [19]. That figure includes counts of all debitage and tools from Block A. Artifacts span elevations of 90.00 to 91.15 m, or 1.15 m of depth. For this part of the site, there is a mean of approximately 6,830 artifacts per 5 cm level.

*Dry Creek, Alaska*

Dry Creek is a multicomponent site situated in a loess deposit mantling a glacio-fluvial terrace of the Nenana River adjacent to the village of Healy, Alaska. The site was first excavated by Powers in the 1970s [23] and later by Graf [24,25]. Three cultural components are preserved in nearly 2 m of alternating silt and sand sediments dating to the Late Pleistocene and Holocene; however, only materials from the two lowermost components were used in the current study. Component 1, found in Loess 2, contains diagnostic artifacts of the Nenana complex, including triangular-shaped finished bifaces, end scrapers made on blades, plano-convex tools, and other tools made of flakes, blades, and cobbles. Component 2 is found in Loess 3 which is stratigraphically separated from underlying Loess 2 by a sterile band of sand. This component contains Denali complex artifacts, including wedge-shaped microblade cores, microblades, burins, and scrapers. Radiocarbon dates (11,530 ± 50 [UCIAMS-135114] and 11,510 ± 40 [Beta-315411]), from a single hearth feature found in the 2-m^2^ block containing the component 1 artifacts used in this study, place their age at about 13,490-13,310 BP. The radiocarbon dates (9480 ± 35 [UCIAMS-135115] and 9460 ± 40 [Beta-315410]) from a nearby hearth feature in component 2 place its age at about 11,070-10,600 BP [24].

Vertical density data are based on all piece-plotted artifacts between N 14 and 16 m and between E 21 and 23 m. Elevations were adjusted by fitting a plane through Component 2 using multiple linear regression. Artifact relative elevations are calculated as the distance above or below that plane. Artifacts span 65.4 cm of depth. For this part of the site, there is a mean of 88.9 piece-plotted artifacts per 5 cm level.

*Gault, Texas*

The Gault site sits along Buttermilk Creek in the Brazos River drainage in Bell County, Texas. It occurs in the Balcones Uplands and is associated with springs and outcrops of Edwards Chert in a zone of riparian vegetation with open grassy meadows and deciduous hardwood trees. The site has a long history of excavation with initial investigations occurring in 1929 [26–29]. Occupations span the entirety of prehistory in the region. Several areas of Gault have been investigated, but our analysis focuses on Area 15. This part of the site includes occupations dating from the Early Paleoindian through Middle Archaic periods. The first occupation is believed to date to 18,500±1,500 BP [28]. Sediments generally fine upward and are believed to largely be alluvial in origin with some colluvial and eolian inputs [27–29]. Artifacts from the Pre-Clovis levels include projectile point fragments that resemble styles from later time periods [28]. The investigators of the site argue that little mixing has affected the deposits because small flakes from the notching of Andice points are only found in the Early and Middle Archaic horizons [28]. However, these artifacts are not confined to a narrow range of elevations but are dispersed vertically over nearly a meter of depth. It has also been argued that there is a 10 cm level of reduced artifact counts between the Clovis and Pre-Clovis components [27,28]**,** but this gap is not readily apparent in published vertical density data from Williams et al. [28]**.** Notably, in those published data, there is a dramatic decline in mean artifact size beneath the Clovis component. There is also a large jump in flake frequency in the Pre-Clovis levels corresponding with increases in natural gravels in bedload or lateral accretion alluvial deposits [28].

We did not have access to piece-plotted artifact data. Therefore, we used vertical density data produced by digitization of Figure S4 from Williams et al. [28]. That figure includes flake counts in 5 cm levels from Strata 1 through 9 spanning elevations of 91.90 to 94.35 m, or 2.45 m of depth. For this part of the site, there is a mean of approximately 698 flakes per 5 cm level.

*Helen Lookingbill, Wyoming*

The Helen Lookingbill site is located in the southern Absaroka Mountains and the Absaroka Plateau, the latter forming the eastern boundary of the Yellowstone Plateau [30]. The site surrounds the spring-fed meadow forming the head of Bain Creek at 2,620 masl, 300 m below Indian Point and 500 m above from where Bain Creek joins Bear Creek. At least three vegetation communities are on the site itself [31]. In the southern part and to the south is sagebrush-grassland with *Artemesia*, *Balsamorhiza*, *Geranium*, and *Pinus*, To the north in the wet meadow are *Ciperaceae*, *Populus*, and *Salix*. To the west under the cliff are grasses (*Graminaea*) and *Populus*. The site provides extensive views to the east and south towards Owl Creek Mountains and Wind River Range respectively.

Two channels enter the meadow. The northern channel created an alluvial fan during the Pleistocene, hence the modern channel splits in two around the fan [32]. The boulder fan extends across the valley forming the substrate of all subsequent deposition. Springs pop up over the low-lying fan, providing sufficient water for beavers to build dams downstream on and below the fan. The result is a bog over lower portions of the fan as well as to the east over Medithermal sediments. The earliest culture bearing strata is the lateral accretion alluvium (Stratum II) of late Pleistocene and early Holocene age with a date of 10,405 ^14^C BP (Beta-28877) near the bottom and 8,525 ^14^C BP (Beta-28116) near the top of the stratum [30]. The three cultural components encased are Haskett, Lovell Constricted, and Pryor Stemmed complexes, from bottom to top respectively. Overlying this is a vertical accretion alluvium of Altithermal age, with dates ranging from 7,360 to 6,460 ^14^C BP (RL-1570a and Beta-61994, respectively) with an Early Plains Archaic component. Medithermal vertical accretion alluvium overlies this with a date of 5,180 ^14^C BP (Beta-61995) with low level cultural activity of Middle and Late Plains Archaic periods [30]. A debris flow of between 1,500 and 3,500 BP (2860 ^14^C BP, Beta-28117) extends over some of the earlier sediments, but stops just short of the sample unit of the current study [30]. Eolian sediments of various types dating to the Pleistocene/Holocene transition and later cover valley slopes, but they are not present in the current area of study.

Vertical density data are based on all piece-plotted artifacts and bone between N 1014 and 1015 m and between E 974 and 976 m. Elevations were adjusted by fitting a plane through all artifacts between elevations 98.7 and 99.1 m using multiple linear regression. Artifact relative elevations are calculated as the distance above or below that plane. Artifacts span 1.7 m of depth. For this part of the site, there is a mean of 74.9 piece-plotted artifacts per 5 cm level.

*Hell Gap, Wyoming*

Hell Gap is a multicomponent Paleoindian site located east of the Haystack Range in the Hartville Uplift of eastern Wyoming around 16 km north of the North Platte River. The Hartville Uplift is a 50 km (N-S) by 25 km (E-W) region of uplifted bedrock capped by Pennsylvanian limestone at the margin of the High Plains and southern Rocky Mountains near Guernsey, WY. The region is characterized by chert and quartzite-rich bedrock exposures forming steep canyons and valleys, sometimes containing ephemeral streams. Valley bottoms in the Uplift serve as depositional basins in which alluvial and colluvial sediments interface to preserve buried archaeological sites that span the entirety of known human occupation of the region. Hell Gap is one such buried archaeological site.

Hell Gap contains at least four buried localities dispersed along a 1 km reach of an ephemeral drainage called Hell Gap Creek [33]. The data used herein are from the University of Wyoming’s excavations at Hell Gap Locality I, which contains the densest concentration of Paleoindian archaeology at the site. Locality I contains no less than eight stratified cultural components spanning ca. 12,800 to 8,500 BP [34]. These components include artifacts of the Goshen, Folsom, Midland, Agate Basin, Hell Gap, Alberta, Cody, and James Allen cultural complexes of the Great Plains and Rocky Mountains [35]. Locality I artifacts are situated within a Late Pleistocene and early Holocene sequence of alluvial sediments (labeled E1-E5, F) derived from overbank flood events of Hell Gap Creek [36]. The lowest cultural bearing sediments of E1 and into E2 date to the Younger Dryas chronozone and possess evidence in the form of Aquoll soils and freshwater mollusks for a wet meadow environment reminiscent of today’s mountain meadows [37,38]. The overlying E units demonstrate gradual drying of the environment into the early Holocene [39]. This Late Pleistocene sediment sequence is present only at the margins of the Hell Gap Valley, where subsequent Holocene cut and fill events have not laterally truncated the deposits [36]. Although Locality I artifacts are vertically-dispersed as a result of bioturbation within buried soils, defined peaks in artifact frequency are readily identifiable as cultural components [34].

Vertical density data were based on all piece-plotted chipped stone, bone, and ocher between N 1481 and 1482 m and between E 1294.5 and 1296.1 m at Locality I. Vertical artifact distributions are based on elevations as measured in the field without adjusting for stratigraphic tilt. Artifacts span 2.46 m of depth. For this part of the site, there is a mean of 12.3 piece-plotted artifacts, bone and ocher per 5 cm level, so the calculated ASI value could be somewhat elevated.

*Holzman South, Alaska*

The Holzman South site was discovered in 2015 on a gently sloping terrace above the confluence of Shaw Creek with the middle Tanana River in interior Alaska. Holzman contains seven stratigraphically sealed cultural components within the upper 170 cm of aeolian silts and sands. The four oldest components date to the late Pleistocene (components 4a, 4b, 5a, and 5b, respectively). The earliest occupation reported thus far is component 5b from sandy loam deposits at 170-160 cm below surface [40] where a female mammoth tusk dated 14,100-14,000 BP along with a bison rib bone dated 13,770-13,520 BP. Chert and basalt lithic flaked debris in contact with charcoal dated 14,150-13,810 BP, among other large mammal bone fragments and ochre. Component 5a consists of flake tools, heavy scrapers, choppers, and an anvil stone associated with a mammoth ivory workstation including two mammoth ivory rods [41] and hearth features with water fowl remains. A burnt Betulaceae (birch or alder) twig from a hearth dated the earliest evidence for human activities at component 5a between 13,590-13,440 BP. No refits have been found between the components and lithic raw material composition differs between components indicating no vertical mixing occurred.

Vertical density data are based on all piece-plotted artifacts and bone between N 185 and 192 m and between E 502 and 506 m. Elevations were adjusted by fitting a plane through Component 5a using multiple linear regression. Artifact relative elevations are calculated as the distance above or below that plane. Artifacts span 1.384 m of depth. For this part of the site, there is a mean of 78.5 piece-plotted artifacts per 5 cm level.

*Owl Ridge, Alaska*

Owl Ridge is a multicomponent site situated in a loess deposit mantling a glacio-fluvial terrace of the Teklanika River, about 25 km northwest of the Dry Creek site and 125 km southwest of Fairbanks, Alaska. The site was first discovered and initially tested in 1976 [42] and revisited and further tested by University of Alaska Fairbanks field crews, directed by Powers, in the late 1970s and early 1980s [43–45]. In 2007-2010 Graf revisited the site and opened a large excavation block [46]. Three cultural components are preserved in 1.25 m of alternating silt and sand sediments dating to the Late Pleistocene and Holocene. Only materials from the two lowermost components were used in the current study. Component 1, found in Paleosol 1 of Loess 1, contains diagnostic artifacts of the Nenana complex, including triangular-shaped finished bifaces and retouched flakes [47]. Component 2 is found in Paleosol 2 of Loess 2 which is stratigraphically separated from underlying Loess 1 by a sterile band of sand. This component contains Denali complex artifacts, including lanceolate bifacial points, scrapers, and a microblade [47]. Two radiocarbon dates (11,340 ± 150 [Beta-11209] and 11,060 ± 60 [AA-86969]) from wood (*Salix* sp.) charcoal found in Paleosol 1 of Loess 1 provide a range of 13,200-12,900 BP to constrain the age of component 1. Thirteen radiocarbon dates ranging from 10,485 ± 25 (UCIAMS-71261) to 10,020 ± 40 (Beta-289378) were obtained on wood (*Salix* sp.) charcoal found in Paleosol 2 of Loess 2 and constrain the age of component 2 to about 12,500-11,600 BP [46] . Spatial analyses of site components found a significant difference in activity locations between components [48], supplementing evidence for stratigraphic integrity of this site.

Vertical density data are based on all piece-plotted chipped stone between N 87 and 89 m and between E 110 and 111 m. Vertical artifact distributions are based on elevations as measured in the field without adjusting for stratigraphic tilt. Artifacts span 77 cm of depth. For this part of the site, there is a mean of 5.3 piece-plotted artifacts per 5 cm level, so the calculated ASI value is likely inflated.

*Shawnee-Minisink, Pennsylvania*

Shawnee-Minisink is a deeply stratified multi-component site at the confluence of the Delaware River and Brodhead Creek in northeast Pennsylvania. The site was first excavated by avocational archaeologist Don Kline and American University in the 1970s [49]. Subsequent excavations of the Clovis level by Gingerich in the early 2000s [50,51] provided precise point provenience data for this study. The Clovis component of the site is buried by ~240 cm of alluvial and eolian deposits [50,51]. The upper portion of the profile contains stratified Late Woodland through Early Archaic Deposits. The Early Archaic deposits terminate at roughly 150 cm below surface [52] and a ~ 90 cm culturally sterile zone (mostly alluvium) is present before reaching the Clovis occupation [51]. The Clovis deposits show minimal evidence post-depositional disturbance with random artifacts orientations and more than 90% of artifacts showing less than 10 degrees of tilt [51]. Extensive lithic refitting of the Clovis component from the American University excavations demonstrate a single occupation of the site [53]. Six radiocarbon dates from preserved hearths that were in close proximity to Clovis projectile points provide an average age estimate of 10,940±15 BP [51,54]; the oldest radiocarbon date is 11,020±30 BP. Below the Clovis component at Shawnee-Minisink, there are several stable surfaces that are nearly identical to the buried surface that contains the Clovis occupation. Testing of these surfaces have shown them to be culturally sterile [51].

Vertical density data were based on all piece-plotted artifacts between N 158.9 and 158.4 m and between E 152.4 and 155.4 m. Elevations were adjusted by fitting a plane through the Clovis component using multiple linear regression. Artifact relative elevations are calculated as the distance above or below that plane. Artifacts span 26.2 cm of depth. For this part of the site, there is a mean of 93.2 piece-plotted artifacts per 5 cm level.

*Swan Point, Alaska*

Swan Point has eight defined occupations that date from the Late Pleistocene to the historic period in over 1 m of loess that caps a small bedrock knoll [55,56] . The site is located in the Shaw Creek drainage of the middle Tanana Valley in central Alaska and was first investigated in 1991 [57]. The oldest occupation, CZ-4b, lies at the base of the loess in grayish brown silt at the interface with intermittent sand and weathered oxidized bedrock rubble [8,58]. A dozen radiocarbon dates (13,800 to 14,500 BP) provide an age of circa 14,150 BP based on hearth charcoal and residue plus collagen from mammoth and horse teeth [9,59]. Component CZ-4b consists of a microblade industry comparable to the Diuktai Culture of Siberia [59,60].

Vertical density data are based on all piece-plotted artifacts and bone between N 90 and 95 m and between E 98 and 99 m. Elevations were adjusted by fitting a plane through all piece-plotted artifacts more than 1.18 m below datum using multiple linear regression. Artifact relative elevations are calculated as the distance above or below that plane. Artifacts span 1.03 m of depth. For this part of the site, there is a mean of 59.3 piece-plotted artifacts per 5 cm level.

**Supplementary References**

1. Johnson DL. Subsurface stone lines, stone zones, artifact-manuport layers, and biomantles produced by bioturbation via pocket gophers (Thomomys bottae). American Antiquity. 1989;54: 370–389.

2. Peacock E, Fant DW. Biomantle formation and artifact translocation in upland sandy soils: An example from the Holly Springs National Forest, north-central Mississippi, U.S.A. Geoarchaeology. 2002;17: 91–114.

3. Mayer JH. Evaluating natural site formation processes in eolian dune sands: A case study from the Krmpotich Folsom site, Killpecker Dunes, Wyoming. Journal of Archaeological Science. 2002;29: 1199–1211.

4. Kennedy C. A Geoarchaeological Site Formation Model at Alm Shelter, Wyoming. Unpublished M.S. Thesis, Department of Anthropology, Utah State University. 2021.

5. Ostahowski BE, Kelly RL, MacDonald DH, Andrefsky W, Yu P-L. Alm Rockshelter Lithic Debitage Analysis: Implications for Hunter-Gatherer Mobility Strategies in the Big Horn Mountains, Wyoming. Lithics in the West. Missoula: University of Montana Press; 2014. pp. 120–141.

6. Holmes CE, Yesner DR. Investigating the Earliest Alaskans: The Broken Mammoth Archaeological Project. Arctic Research of the United States. 1992;6: 6–9.

7. Holmes CE. Broken Mammoth. American Beginnings: The Prehistory and Paleoecology of Beringia. Chicago: University of Chicago Press; 1996. pp. 312–318.

8. Dilley TE. Late Quaternary Loess Stratigraphy, Soils, and Environments of the Shaw Creek Flats Paleoindian sites, Tanana Valley, Alaska. Ph.D. Dissertation, The University of Arizona. 1998.

9. Potter BA, Holmes CE, Yesner DR. Technology and economy among the earliest prehistoric foragers in interior eastern Beringia. In: Graf KE, Ketron CV, Waters MR, editors. Paleoamerican odyssey. College Station, Texas: Texas A&M University Press; 2013. pp. 81–103.

10. Butler BR. Contributions to the Prehistory of the Columbia Plateau: A Report on Excavations in the Palouse and Craig Mountain Sections. Pocatello, Idaho: Occasional Papers of the Idaho State College Museum, Number 9; 1962.

11. Davis LG, Schweger CE. Geoarchaeological context of late Pleistocene and early Holocene occupation at the Cooper’s Ferry site, western Idaho, USA. Geoarchaeology. 2004;19: 685–704. doi:https://doi.org/10.1002/gea.20020

12. Davis LG, Madsen DB, Becerra-Valdivia L, Higham T, Sisson DA, Skinner SM, et al. Late Upper Paleolithic occupation at Cooper’s Ferry, Idaho, USA, ~16,000 years ago. Science. 2019;365: 891–897.

13. Davis LG, Nyers AJ, Willis SC. Context, provenance and technology of a western stemmed tradition artifact cache from the Cooper’s Ferry Site, Idaho. American Antiquity. 2014;79: 596–615.

14. Davis LG, Madsen DB, Sisson DA, Izuho M. Response to Review of “Late Upper Paleolithic occupation at Cooper’s Ferry, Idaho, USA,~16,000 years ago” by Fiedel et al. PaleoAmerica. 2021;7: 43–52.

15. Fiedel SJ, Potter BA, Morrow JE, Faught MK, Haynes CV Jr, Chatters JC. Pioneers from Northern Japan in Idaho 16,000 Years Ago? A Critical Evaluation of the Evidence from Cooper’s Ferry. PaleoAmerica. 2020;7: 28–42.

16. Driese SG, Nordt LC, Waters MR, Keene JL. Analysis of Site Formation History and Potential Disturbance of Stratigraphic Context in Vertisols at the Debra L. Friedkin Archaeological Site in Central Texas, USA. Geoarchaeology. 2013;28: 221–248. doi:10.1002/gea.21441

17. Jennings TA. Clovis, Folsom, and Midland components at the Debra L. Friedkin site, Texas: context, chronology, and assemblages. Journal of Archaeological Science. 2012;39: 3239–3247. doi:10.1016/j.jas.2012.05.007

18. Jennings TA, Waters MR. Pre-Clovis Lithic Technology at the Debra L. Friedkin Site, Texas: Comparisons to Clovis through Site-Level Behavior, Technological Trait-List, and Cladistic Analyses. American Antiquity. 2014;79: 25–44. doi:10.7183/0002-7316.79.1.25

19. Waters MR, Keene JL, Forman SL, Prewitt ER, Carlson DL, Wiederhold JE. Pre-Clovis projectile points at the Debra L. Friedkin site, Texas- Implications for the Late Pleistocene peopling of the Americas. Science advances. 2018;4: eaat4505.

20. Waters MR, Forman SL, Jennings TA, Nordt LC, Driese SG, Feinberg JM, et al. The Buttermilk Creek Complex and the Origins of Clovis at the Debra L. Friedkin Site, Texas. Science. 2011;331: 1599–1603.

21. Lindquist AK, Feinberg JM, Waters MR. Rock magnetic properties of a soil developed on an alluvial deposit at Buttermilk Creek, Texas, USA. Geochemistry, Geophysics, Geosystems. 2011;12.

22. Morrow JE, Fiedel SJ, Johnson DL, Kornfeld M, Rutledge M, Wood WR. Pre-Clovis in Texas? A critical assessment of the “Buttermilk Creek Complex.” Journal of Archaeological Science. 2012;39: 3677–3682.

23. Powers WR, Guthrie RD, Hoffecker JF. Dry Creek: Archaeology and paleoecology of a late Pleistocene Alaskan hunting camp. Goebel T, editor. College Station: Texas A&M University Press; 2017.

24. Graf KE, DiPietro LM, Krasinski KE, Gore AK, Smith HL, Culleton BJ, et al. Dry Creek Revisited: New Excavations, Radiocarbon Dates, and Site Formation Inform on the Peopling of Eastern Beringia. Am antiq. 2015;80: 671–694. doi:10.7183/0002-7316.80.4.671

25. Graf KE, DiPietro LM, Krasinski K, Culleton BJ, Kennett DK, Gore AK, et al. New geoarchaeology and geochronology at Dry Creek. In: Powers WR, Guthrie RD, Hoffecker JF, Goebel T, editors. Dry Creek: Archaeology and Paleoecology of a late Pleistocene Alaskan hunting camp. College Station, Texas: Texas A&M University Press; 2017. pp. 219–260.

26. Collins MB. The Gault site, Texas and Clovis research. Athena Review. 2002;3: 31–41.

27. Gilmer A. Geoarchaeological Investigations of Site Formation Processes in Area 15 at the Gault Site, Bell County, Texas. Unpublished M.A. Thesis, Texas State University. 2013.

28. Williams TJ, Collins MB, Rodrigues K, Rink WJ, Velchoff N, Keen-Zebert A, et al. Evidence of an early projectile point technology in North America at the Gault Site, Texas, USA. Science Advances. 2018;4: eaar5954.

29. Waters MR, Pevny CD, Carlson DL. Clovis Lithic Technology: Investigation of a Stratified Workshop at the Gault Site, Texas. College Station: Texas A&M Press; 2011.

30. Kornfeld M, Larson ML, Rapson DJ, Frison GC. 10,000 years in the Rocky Mountains: The Helen Lookingbill site. Journal of Field Archaeology. 2001;28: 307–324.

31. Scott-Cummings L. Appendix A: Pollen analysis of the Helen Lookingbill site (48FR308), Wyoming. In: Larson ML, Kornfeld M, Rapson DJ, editors. High Altitute Hunter-Gatehrer Adaptations in the Middle Rocky Mountains: 1988-1994 Investigations. Laramie, WY: Technical Report No. 4, Department of Anthropology, University of Wyoming; 1995.

32. Miller JC. Appendix B: Geology of the Helen Lookingbill site (48FR308), Fremont County, Wyoming. In: Larson ML, Kornfeld M, Rapson DJ, editors. High Altitute Hunter-Gatehrer Adaptations in the Middle Rocky Mountains: 1988-1994 Investigations. Laramie, WY: Technical Report No. 4, Department of Anthropology, University of Wyoming; 1995.

33. Kornfeld M, Larson, M. L. JI. Introduction to Hell Gap and History of Research. In: Larson ML, Kornfeld M, Frison GC, editors. Hell Gap: A Stratified Paleoindian Campsite at the Edge of the Rockies. Salt Lake City: University Of Utah Press; 2009. pp. 3–13.

34. Pelton SR, Kornfeld M, Larson ML, Minckley T. Component age estimates for the Hell Gap Paleoindian site and methods for chronological modeling of stratified open sites. Quaternary Research. 2017;88: 234–247.

35. Irwin-Williams C, Irwin H, Agogino G, Haynes CV. Hell Gap: Paleo-indian occupation on the High Plains. Plains Anthropologist. 1973;18: 40–53.

36. Haynes CV Jr. Geochronology. In: Larson ML, Kornfeld M, Frison GC, editors. Hell Gap: A Stratified Paleoindian Campsite at the Edge of the Rockies. Salt Lake City: University Of Utah Press; 2009. pp. 39–52.

37. Jass CN, Mead JI. Gastropods and their Paleoenvironmental Implications. In: Larson ML, Kornfeld M, Frison GC, editors. Hell Gap: A Stratified Paleoindian Campsite at the Edge of the Rockies. Salt Lake City: University Of Utah Press; 2009. pp. 99–102.

38. Reider R. Soil Development. In: Larson ML, Kornfeld M, Frison GC, editors. Hell Gap: A Stratified Paleoindian Campsite at the Edge of the Rockies. Salt Lake City: University Of Utah Press; 2009. pp. 53–71.

39. Fredlund G. Phytolith evidence for vegetation and climate change during the Pleistocene-Holocene transition. In: Larson ML, Kornfeld M, Frison GC, editors. Hell Gap: A Stratified Paleoindian Campsite at the Edge of the Rockies. Salt Lake City: University Of Utah Press; 2009. pp. 90–98.

40. Wygal BT, Krasinski KE, Holmes CE, Crass BA. Holzman South: A Late Pleistocene Archaeological Site along Shaw Creek, Tanana Valley, Interior Alaska. PaleoAmerica. 2018;4: 90–93.

41. Wygal BT, Krasinski KE, Holmes CE, Crass, BA, Smith KM. Mammoth ivory rods in eastern Beringia: Earliest in North America. American Antiquity. in press; 1–21.

42. Plaskett DC. Preliminary report: A Cultural resources survey in an area of the Nenana and Teklanika rivers of central Alaska. Unpublished manuscript; 1976.

43. Hoffecker JF. Applied geomorphology and archaeological survey strategy for sites of Pleistocene age: An example from central Alaska. Journal of Archaeological Science. 1988;15: 683–713.

44. Hoffecker JF, Powers WR, Phippen PG. Owl ridge. In: West FH, editor. American beginnings: The Prehistory and Paleoecology of Beringia. Chicago: University of Chicago Press; 1996. pp. 353–356.

45. Phippen PG. Archaeology at Owl Ridge: A Pleistocene‐Holocene boundary age site in central Alaska. Unpublished M.A. Thesis, Department of Anthropology, University of Alaska, Fairbanks. 1988.

46. Graf KE, Gore AK, Melton JA, Marks T, DiPietro L, Goebel T, et al. Recent excavations at Owl Ridge, interior Alaska: Site stratigraphy, chronology, and site formation and implications for late Pleistocene archaeology and peopling of eastern Beringia. Geoarchaeology. 2020;35: 3–26.

47. Gore AK, Graf KE. Technology and Human Response to Environmental Change at the Pleistocene-Holocene Boundary in Eastern Beringia: A View from Owl Ridge, Central Alaska. In: Robinson E, Sellet F, editors. Lithic Technological Organization and Paleoenvironmental Change. Springer; 2018. pp. 203–234.

48. Puckett NN, Graf KE. Understanding Space at Owl Ridge, Central Alaska. Diversity in Open Air Site Structure across the Pleistocene/Holocene Boundary. In: Carlson KC, Bement LC, editors. Diversity in Open Air Site Structure across the Pleistocene/Holocene Boundary. Boulder: University Press of Colorado; 2022.

49. McNett CW. Shawnee Minisink: a stratified Paleoindian–Archaic site in the upper Delaware Valley of Pennsylvania. Academic Press; 1985.

50. Gingerich JAM. Shawnee-Minisink Revisited: Re-Evaulating the Paleoindian Occupation. Department of Anthropology. University of WyomingEditor. 2007.

51. Gingerich JAM. Revisiting the Shawnee-Minisink site. In: Gingerich JAM, editor. The Eastern Fluted Point Tradition. Salt Lake City: University of Utah Press; 2013. pp. 218–257.

52. McMillan BA. A Technological Analysis of the Early Archaic. In: McNett CW, editor. Shawnee Minisink. New York: Academic Press; 1985. pp. 261–319.

53. Gingerich JAM. Understanding Refitting and Artifact Distributions in Spatial Context: A Paleoindian Case Study. Intrasite Spatial Analysis of Mobile and Semisedentary Peoples. In: Clark AE, Gingerich JAM, editors. Intrasite Spatial Analysis of Mobile and Semisedentary Peoples. Salt Lake City: University Of Utah Press; 2022. pp. 53–69.

54. Gingerich JAM. Down to seeds and stones: A new look at the subsistence remains from Shawnee-Minisink. American Antiquity. 2011;76: 127–144.

55. Holmes CE. The Taiga period: Holocene archaeology of the northern boreal forest, Alaska. Alaska Journal of Anthropology. 2008;6: 69–81.

56. Hirasawa Y, Holmes CE. The relationship between microblade morphology and production technology in Alaska from the perspective of the Swan Point site. Quaternary International. 2017;442: 104–117.

57. Holmes CE, VanderHoek R, Dilley TE. Swan Point. In: West FH, editor. American Beginnings. Chicago: University of Chicago Press; 1996. pp. 319–322.

58. Kielhofer J, Miller C, Reuther J, Holmes C, Potter B, Lanoë F, et al. The micromorphology of loess-paleosol sequences in central Alaska: A new perspective on soil formation and landscape evolution since the Late Glacial period (c. 16,000 cal yr BP to present). Geoarchaeology. 2020;35: 701–728.

59. Gómez Coutouly YA, Holmes CE. The microblade industry from Swan Point CZ4b: Technological and cultural implications from the earliest human occupation in Alaska. American Antiquity. 2018;83: 735–752.

60. Holmes CE. The Beringian and transitional periods in Alaska: Technology of the East Beringian tradition as viewed from Swan Point. In: Goebel T, Buvit I, editors. From the Yenisei to the Yukon: Interpreting Lithic Assemblage Variability in Late Pleistocene/Early Holocene Beringia. College Station, Texas: Texas A&M University Press; 2011. pp. 179–191.
